# Supplementary material for: Functional genomics analysis of developing zebrafish and human endoderm reveals highly conserved cis-regulatory modules acting during vertebrate organogenesis
Source: Genome Res. Author manuscript; Available in PMC 2026 May 6. (PMC7619044; doi:10.1101/gr.280838.125)
Supplement: Supplementary Materials [file EMS213377-supplement-Supplementary_Materials.pdf]

Supplemental Material

Functional genomics analysis of developing zebrafish and human endoderm reveals highly conserved *cis*-regulatory modules acting during vertebrate organogenesis

Daniela M. Riley, Randa Elsayed, Mark D. Walsh, Simaran Johal, Ying Lin, Harry Walton, Till Bretschneider, Sascha Ott and Andrew C. Nelson

Supplemental Figure S1. Representative example plots of FACS separation of sox17E, sox17M and sox17N populations at 28 hpf and 48 hpf.

Supplemental Figure S2. Biological replicate RNA-seq samples from 28 hpf sorted cell populations cluster based on sample identity, with the sox17E population enriched for endoderm marker expression.

Supplemental Figure S3. *Tg(sox17:GFP)* 48 hpf embryos show expression in the median fin fold, and muscle cells in the tail.

Supplemental Figure S4. Biological replicates of sorted populations cluster together based on ATAC-seq peak scores.

Supplemental Figure S5. ATAC-seq peaks show similar read densities between biological replicates.

Supplemental Figure S6. Venn diagrams indicating overlap of ATAC-seq peaks identified per cell population per stage.

Supplemental Table 2. Number of DARs between sorted cell populations at 28 and 48 hpf.

Supplemental Table 3. Number of DARs between 28 and 48 hpf in the sorted cell populations.

Supplemental Figure S7. Genomic distribution of DARs relative to gene annotations.

Supplemental Figure S8. Tracks in Figure 2 zoomed out and rescaled to the strongest local peak.

Supplemental Figure S9. GFP+ cells in the posterior notochord of *sox17:GFP* arise from *sox32*-dependent cells.

Supplemental Figure S10. *Sox17*N and *sox17*M populations are not enriched for endoderm marker promoter accessibility compared to *sox17*E.

Supplemental Figure S11. *sox17:GFP* expression in the posterior lateral line is sensitive to RA treatment.

Supplemental Table 11. Number of H3K27ac peaks called in each cell population drops with more stringent q-values.

Supplemental Figure S12. Human endoderm H3K27ac peaks and/or zebrafish *sox17E>sox17M* DARs overlap with 9% of zebrafish/human HCNEs.

Supplemental Table 13. Number of HCNEs overlapping zebrafish *sox17*E>*sox17*M DARs and H3K27ac peaks in anterior-posterior patterned endoderm cell populations derived from hESCs.

Supplemental Figure S13. Overlap of HCNEs with *sox17E>sox17M* DARs and H3K27ac.

Supplemental Figure S14. DNA-binding terms are found in top GO molecular function terms enriched in genes near endodermal HCNEs.

Supplemental Figure S15. There is enrichment for embryonic development and transcription regulation in the top GO biological process terms enriched in genes near endodermal HCNEs.

Supplemental Figure S16. There is enrichment for transcription terms in the top GO cellular component terms enriched in genes near endodermal HCNEs.

Supplemental Figure S17. A range of mouse phenotypes are enriched in endodermal HCNE associated genes.

Supplemental Figure S18. There is enrichment for a variety of phenotypes in the top human phenotype terms enriched in genes near endodermal HCNEs.

Supplemental Figure S19. Clustering of known and *de novo* motifs corresponding to Figure 5.

Supplemental Figure S20. FOXA2 binds at HCNEs during hESC to pancreatic endoderm differentiation, followed by subsequent PTF1A binding.

Supplemental Figure S21. Human and zebrafish endoderm HCNEs show consistent coincidence of transcription factor binding sites (TFBSs) but with limited grammatical consistency either within or between species.

Supplemental Figure S22. Location of orthologous human-zebrafish HCNEs exhibiting consistent 12 bp spacing of bHLH and homeodomain TFBSs indicated in Figure 6.

Supplemental Figure S23. Co-expression in developing zebrafish endoderm of homeodomain-bHLH TF pairs depicted in Figure 6.

Supplemental Figure S24. HCNEs in introns 4 and 5 of zebrafish *hnf1ba* and human *HNF1B* are not conserved in zebrafish *hnf1bb*.

Supplemental Figure S25. PDX1 binds the intron 5 *HNF1B* HCNE in human pancreatic progenitors.

Supplemental Figure S26. *Hnf1ba* is co-expressed with TF genes whose TFBSs co-occur in intron 4 HCNEs.

Supplemental Table 14. Coordinates of putative enhancers and HCNEs of *hnf1ba* studied.

Supplemental Table 15. Table showing where enhancers drove reporter expression in developing zebrafish at 48 hpf.

Supplemental Figure S27. Putative *hnf1ba* enhancers show reporter expression in multiple cell types including in the brain and neural tube.

Supplemental Figure S28. Consistent expression in the hindbrain driven by *hnf1ba* putative enhancers.

Supplemental Figure S29. *Hnf1ba* HCNEs from human and zebrafish both drive expression in the hindbrain.

Supplemental Figure S30. Functional genomics analysis of the broader *HNF1B* genomic neighbourhood indicates binding of key pancreatic transcription factors at non-HCNE CRMs.

Supplemental Figure S31. *Sox17*E ATAC-seq analysis indicates potential CRMs upstream and in intron 8 of *hnf1ba* that could regulate endodermal expression.

Supplemental Table 16. Numbers of reads per sample passing filtering and used in analyses.

Supplemental Table 17. Primers used in this study.

Supplemental Methods

Supplemental Tables 1, 4-10 & 12, provided as a separate Excel file.

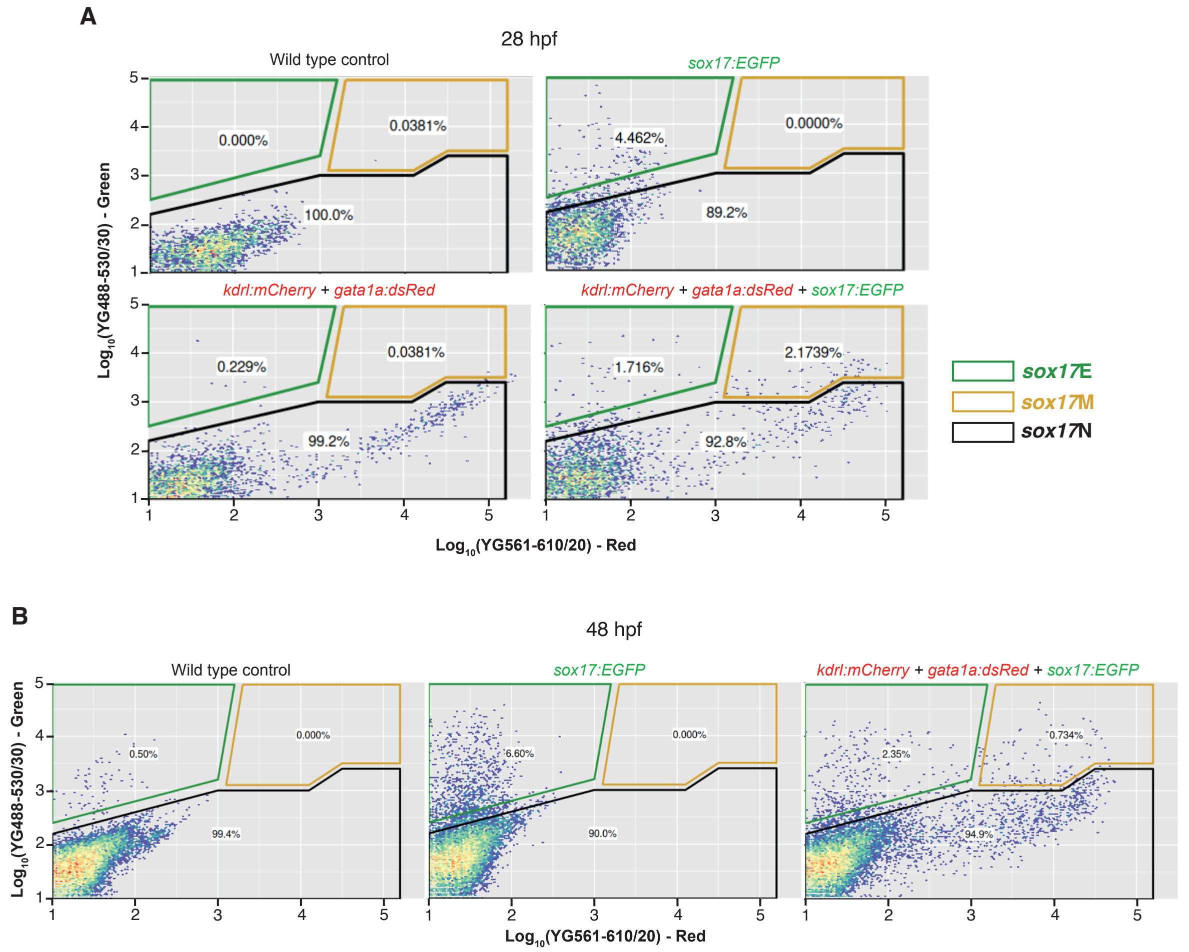

Supplemental Figure S1: Representative example plots of FACS separation of sox17**E, *****sox17*****M and *****sox17*****N populations at 28 hpf (A) and 48 hpf (B)*.**

Percentage of singlet events in each gate are indicated. Gates were set using negative (wild type) and single-colour controls to minimise false positive selection of cells. Cells were then sorted from the triple transgenic reporters *kdrl:mCherry* + *gata1a:dsRed* + *sox17:GFP*. Noting the general increase in green fluorescence in *sox17:GFP* cells compared to wild type, *sox17*E cell selection was therefore deliberately conservative in order to minimise erroneous selection of cells potentially due to leaky transgene expression. Higher percentages of cells in the green gate in the *sox17:GFP* only control compared to the triple transgenic reporter are likely due to homozygous cells in the *sox17:GFP* only control while all triple transgenic cells are necessarily heterozygous. We note that while at 48 hpf a small percentage of cells (0.5%) exhibit autofluorescence, we nevertheless achieve 6-fold enrichment over background in sorts from the triple transgenic reporters.

Also see Figures 1 and 2, indicating strong enrichment for endoderm-specific transcripts, and accessibility at endoderm-specific marker genes in the *sox17*E population over *sox17*N, indicating good enrichment for endoderm.

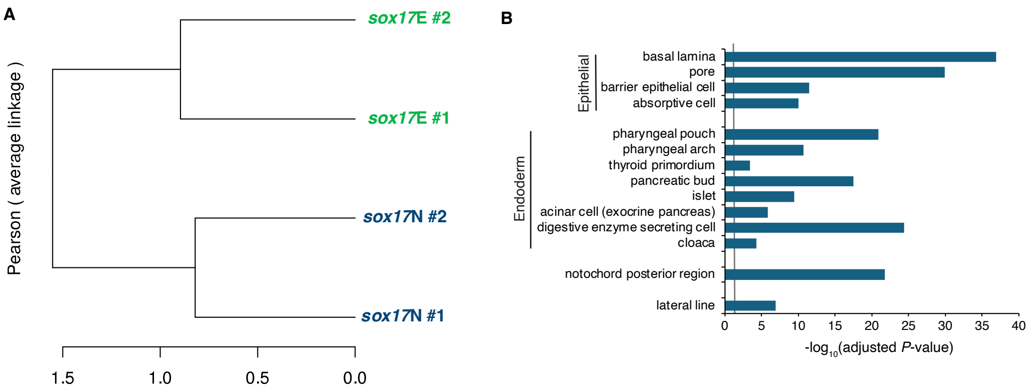

Supplemental Figure S2: Biological replicate RNA-seq samples from 28 hpf sorted cell populations cluster based on sample identity, with the sox17E population enriched for endoderm marker expression. *(A) Pearson correlation based on the top 75% most highly expressed genes (default in iDEP v2.01). (B) fishEnrichr Anatomy GeneRIF Predicted Z-score analyses of all genes significantly upregulated in sox17E>sox17N (see Supplemental Table 1). Selected terms are presented categorised according to general epithelial identity characteristic of endodermal cells at 28 hpf, as well as more specific endoderm anatomical terms. Note enrichment for posterior notochord, consistent with transdifferentiation of Kupffer's vesicle, and also enrichment for lateral line markers. The grey line indicates -log*$_{10}$*(adjusted P-value) equivalent to P = 0.05.*

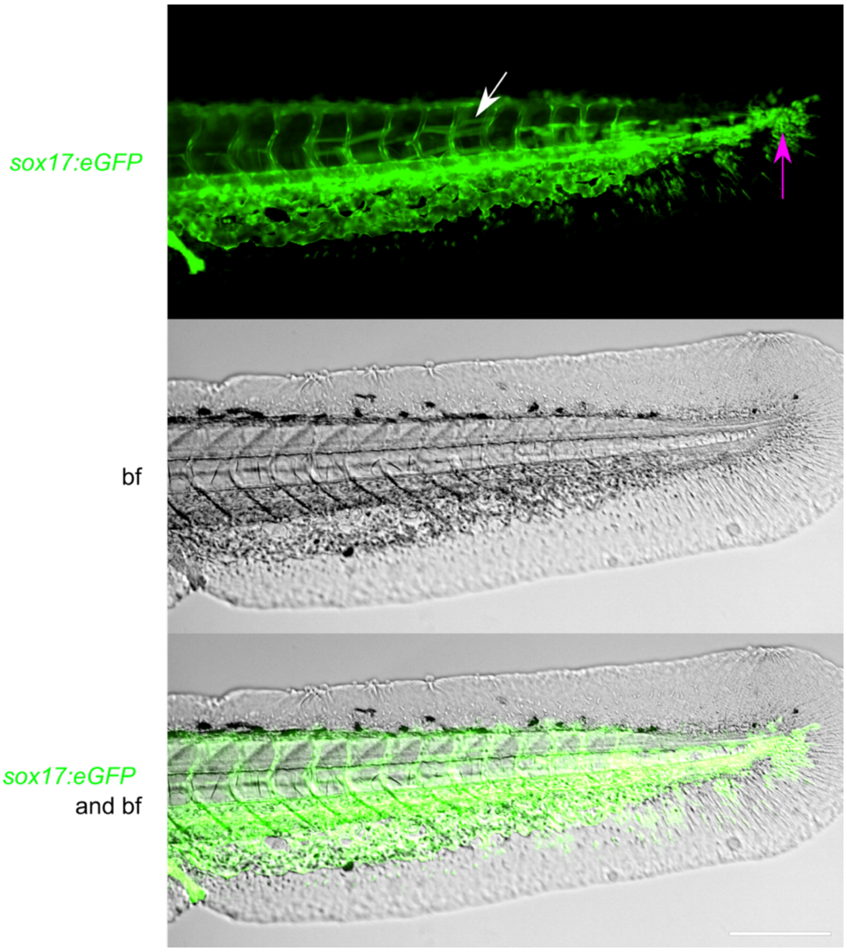

Supplemental Figure S3: Tg(sox17:GFP) 48 hpf embryos show expression in the median fin fold, and muscle cells in the tail. *Images of lateral orientated 48 hpf embryos. Math Log function has been applied to the images. White arrow is pointing at muscle cells that are eGFP positive. Pink arrow points at GFP in the median fin fold cells. White scale bar in composite image represents 500 µm.*

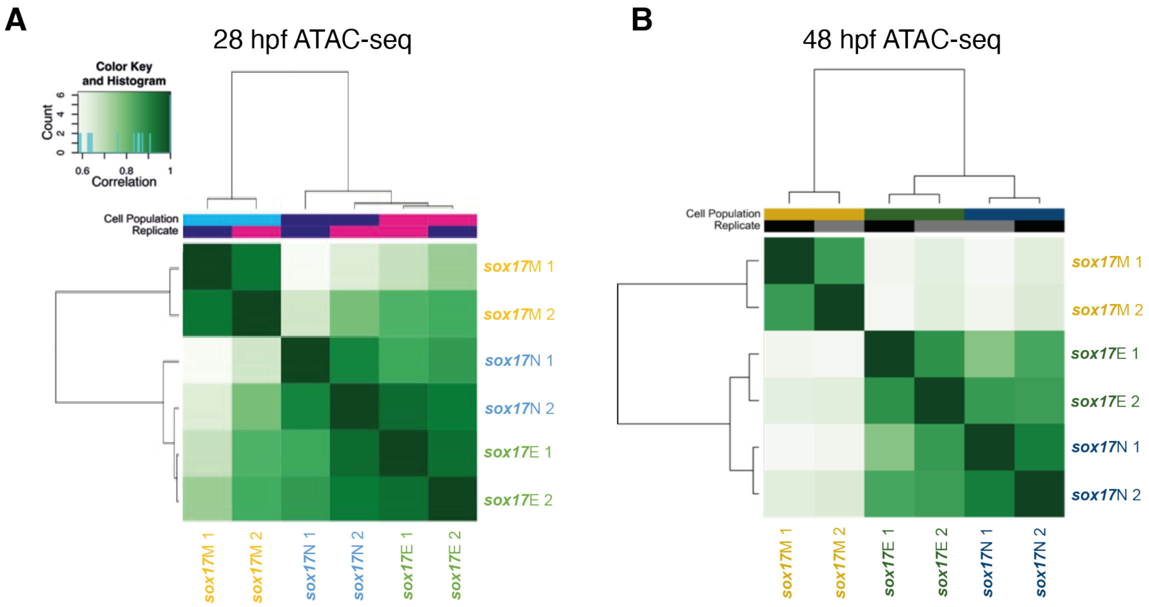

Supplemental Figure S4. Biological replicates of sorted populations cluster together based on ATAC-seq peak scores. *Pearson correlation heatmap based on MACS2 scores for ATAC-seq data at 28 hpf (A) and 48 hpf (B).*

A

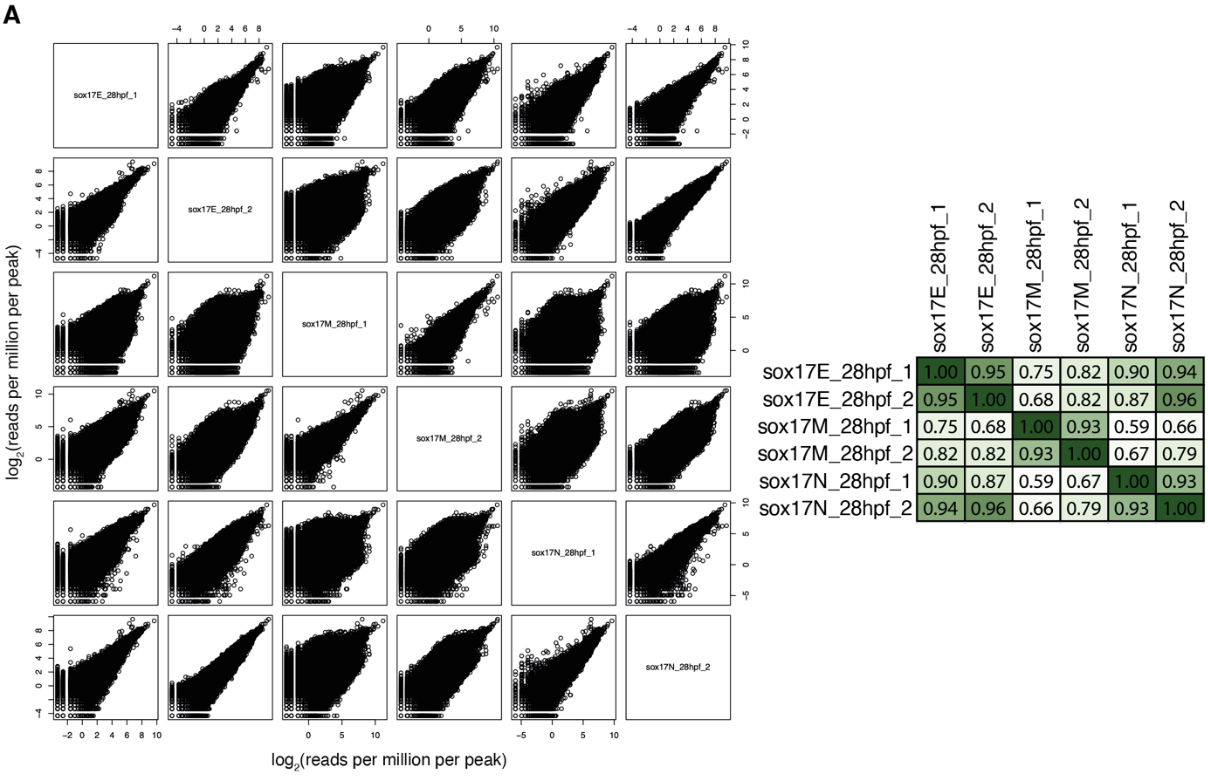

	sox17E_28hpf_1	sox17E_28hpf_2	sox17M_28hpf_1	sox17M_28hpf_2	sox17N_28hpf_1	sox17N_28hpf_2
sox17E_28hpf_1	1.00	0.95	0.75	0.82	0.90	0.94
sox17E_28hpf_2	0.95	1.00	0.68	0.82	0.87	0.96
sox17M_28hpf_1	0.75	0.68	1.00	0.93	0.59	0.66
sox17M_28hpf_2	0.82	0.82	0.93	1.00	0.67	0.79
sox17N_28hpf_1	0.90	0.87	0.59	0.67	1.00	0.93
sox17N_28hpf_2	0.94	0.96	0.66	0.79	0.93	1.00

B

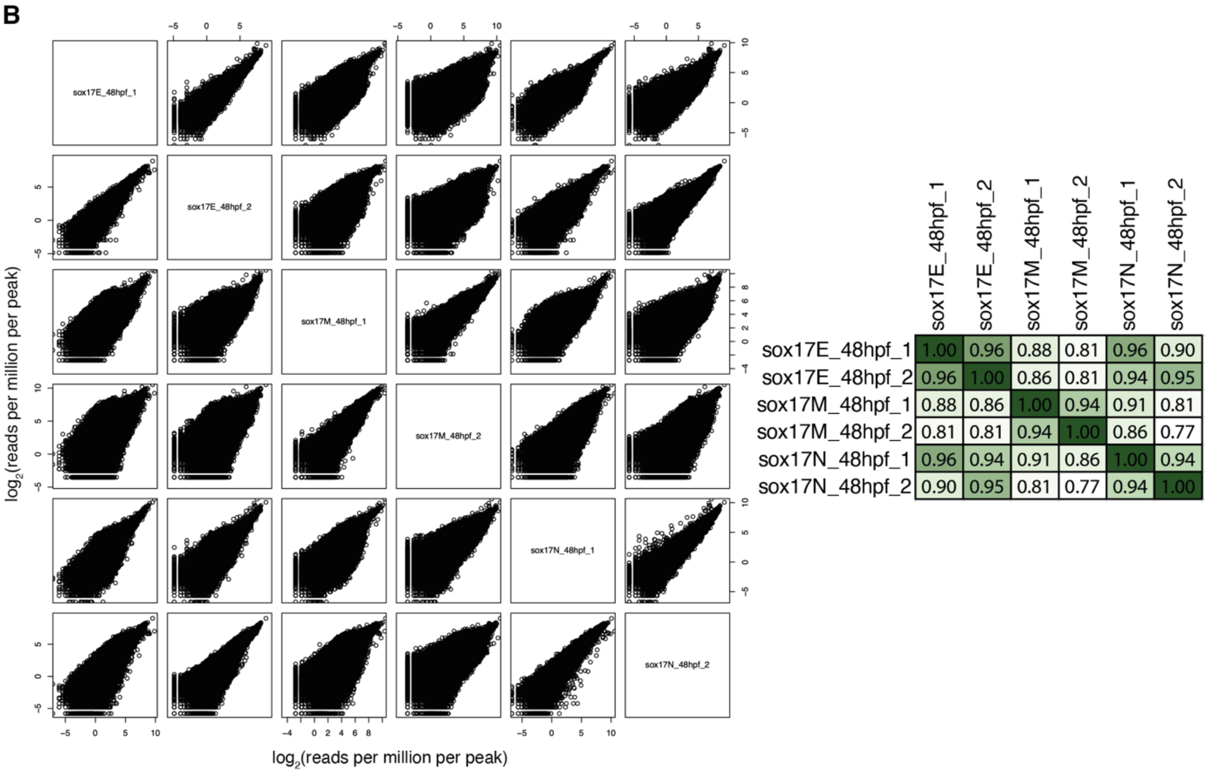

	sox17E_48hpf_1	sox17E_48hpf_2	sox17M_48hpf_1	sox17M_48hpf_2	sox17N_48hpf_1	sox17N_48hpf_2
sox17E_48hpf_1	1.00	0.96	0.88	0.81	0.96	0.90
sox17E_48hpf_2	0.96	1.00	0.86	0.81	0.94	0.95
sox17M_48hpf_1	0.88	0.86	1.00	0.94	0.91	0.81
sox17M_48hpf_2	0.81	0.81	0.94	1.00	0.86	0.77
sox17N_48hpf_1	0.96	0.94	0.91	0.86	1.00	0.94
sox17N_48hpf_2	0.90	0.95	0.81	0.77	0.94	1.00

Supplemental Figure S5. ATAC-seq peaks show similar read densities between biological replicates. *Pairwise XY scatter plot matrix of log_2 transformed reads per million per peak comparing all biological samples at 28 hpf (left), and corresponding Pearson correlation matrix based on read counts for ATAC-seq data at 28 hpf (A) and 48 hpf (B).*

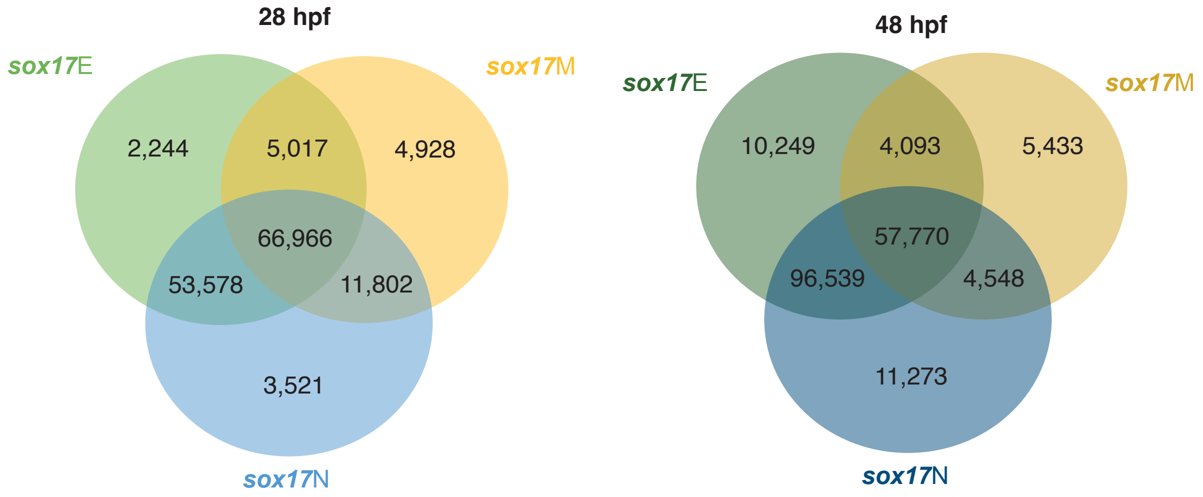

Supplemental Figure S6. Venn diagrams indicating overlap of ATAC-seq peaks identified per cell population per stage. *Depicted are numbers of unique ATAC-seq peaks used by DiffBind that were called as significant peaks in the different populations by MACS2 prior to differential accessibility analysis. Note that peaks can be shared by the populations (i.e. be detectable), but show markedly different degrees of accessibility. Similarly, a peak can narrowly meet a threshold to be called in one population, narrowly miss the threshold in another population and yet not be significantly different between the two populations. While these numbers are informative, the DARs depicted in Figure 2 provide a more robust measure of chromatin accessibility differences between populations.*

Supplemental Table 2: Number of DARs between sorted cell populations at 28 and 48 hpf

**Cell populations compared**	**Comparison type**	**Number of regions (DARs) exhibiting greater accessibility per timepoint**			
		28 hpf		48 hpf	
		FDR<0.05	FDR <0.01	FDR <0.05	FDR <0.01
Sox17M vs Sox17E	Sox17M > Sox17E	10,300	6,267	7,823	5,500
	Sox17E > Sox17M	23,344	12,898	46,703	28,172
Sox17N vs Sox17E	Sox17N > Sox17E	1,102	107	6,209	1,557
	Sox17E > Sox17N	599	91	5,692	2,542
Sox17N vs Sox17M	Sox17N > Sox17M	29,133	19,557	60,282	41,719
	Sox17M > Sox17N	12,367	8,521	8,741	5,293

Supplemental Table 3: Number of DARs between 28 and 48 hpf in the sorted cell populations

**Cell populations compared**	**Comparison type**	**Number of regions (DARs) exhibiting greater accessibility**	
		FDR <0.05	FDR <0.01
**Sox17E**	28 hpf > 48 hpf	985	395
	48 hpf > 28 hpf	1,707	22
**Sox17M**	28 hpf > 48 hpf	4,828	2,317
	48 hpf > 28 hpf	1,090	478
**Sox17N**	28 hpf > 48 hpf	5,788	3,100
	48 hpf > 28 hpf	4,136	1,264

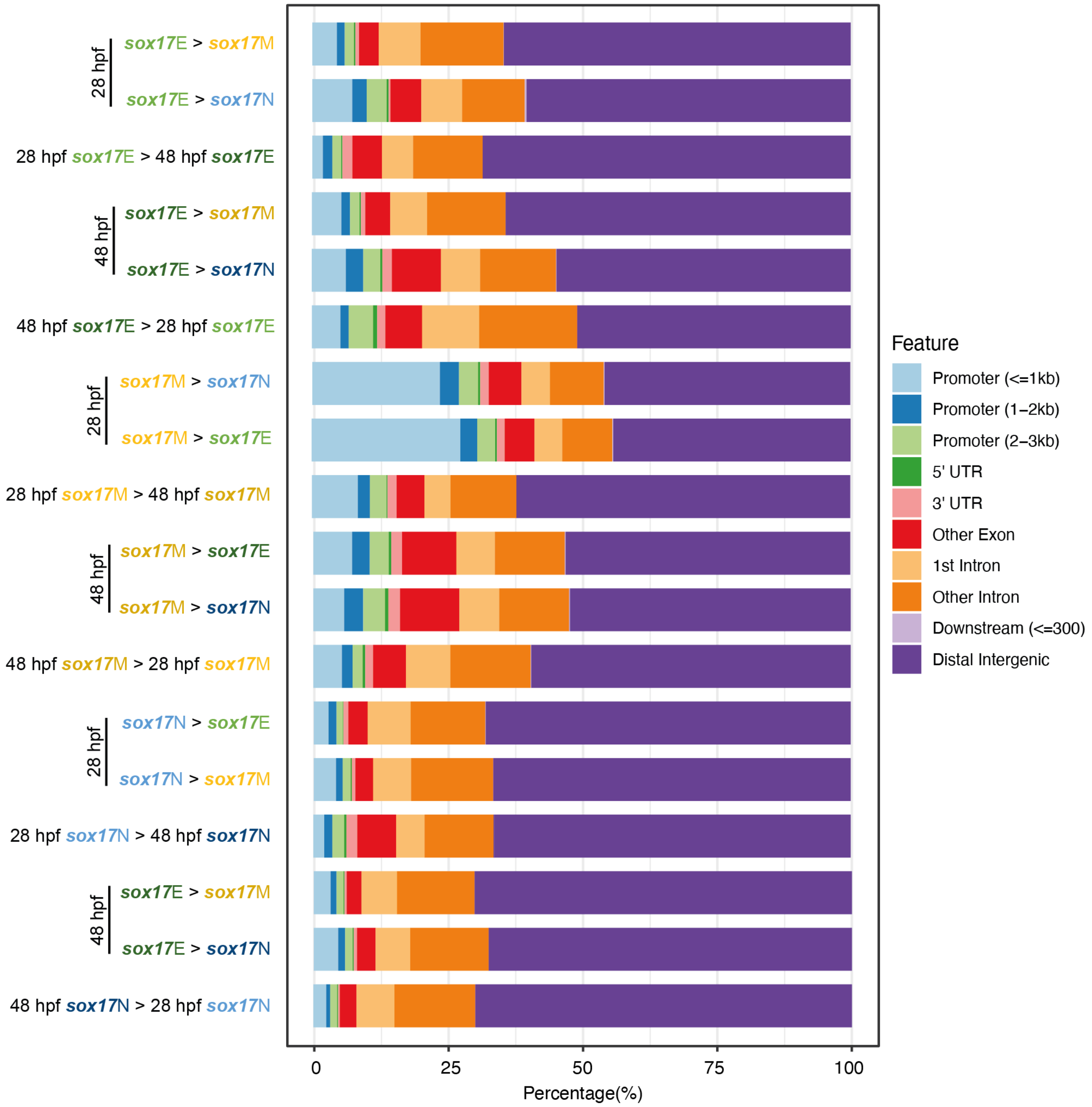

Supplemental Figure S7. Genomic distribution of DARs relative to gene annotations. *DARs from all pairwise comparisons were related to zebrafish danRer11 gene annotations using ChIPseeker. The percentage DARs in each location bin are indicated.*

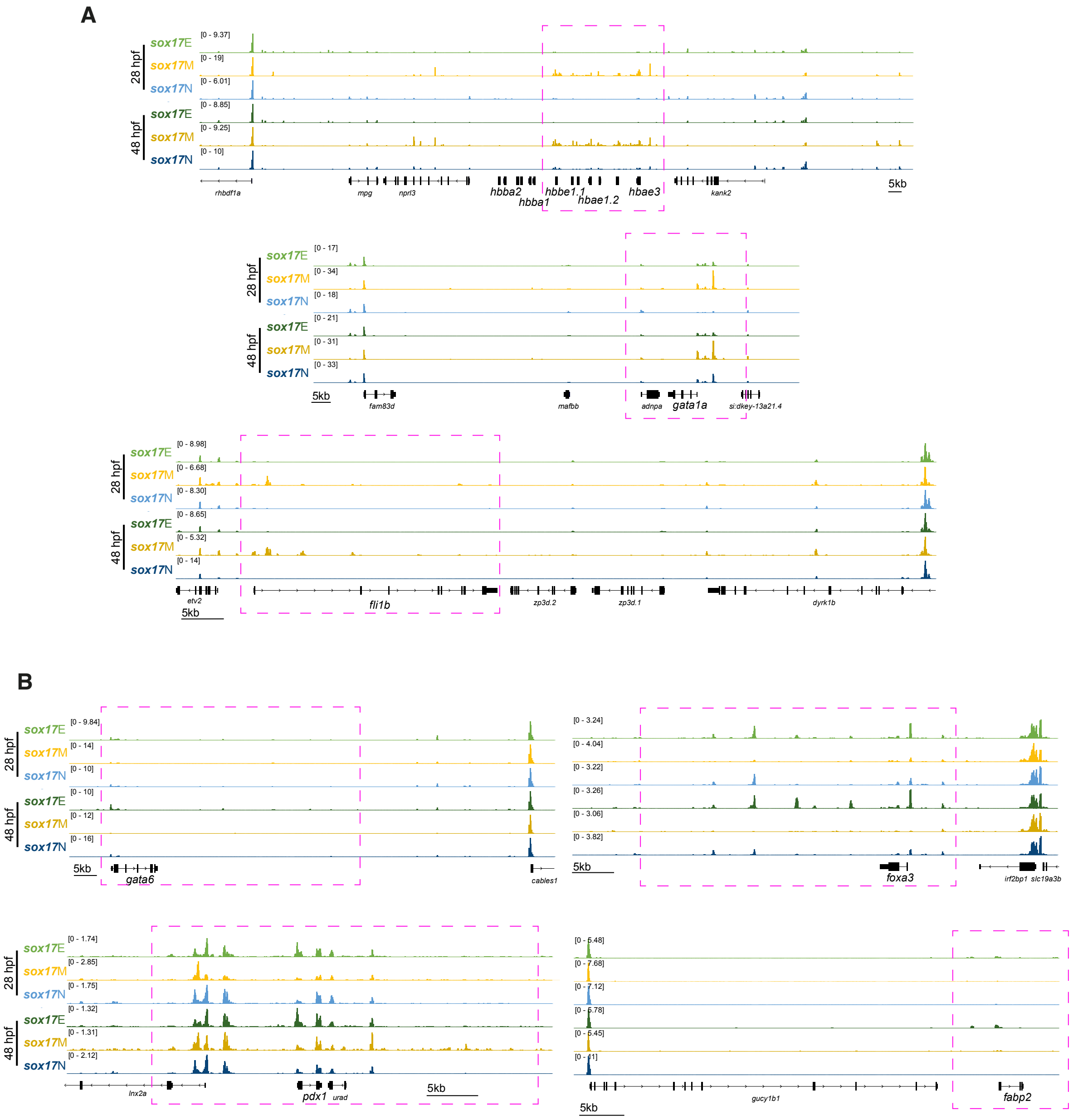

Supplemental Figure S8. Tracks in Figure 2 zoomed out and rescaled to the strongest local peak. *(A) Tracks shown in Fig. 2C. (B) Tracks shown in Fig. 2D. Pink boxes indicate the genomic coordinates displayed in Figure 2. Scale ranges in CPM are shown for each individual track. Every differentially accessible region (DAR) annotated in red boxes in Figure 2 is clearly identifiable in this figure where each track is scaled to the largest local peak, suggesting the differences highlighted in Figure 2 are real.*

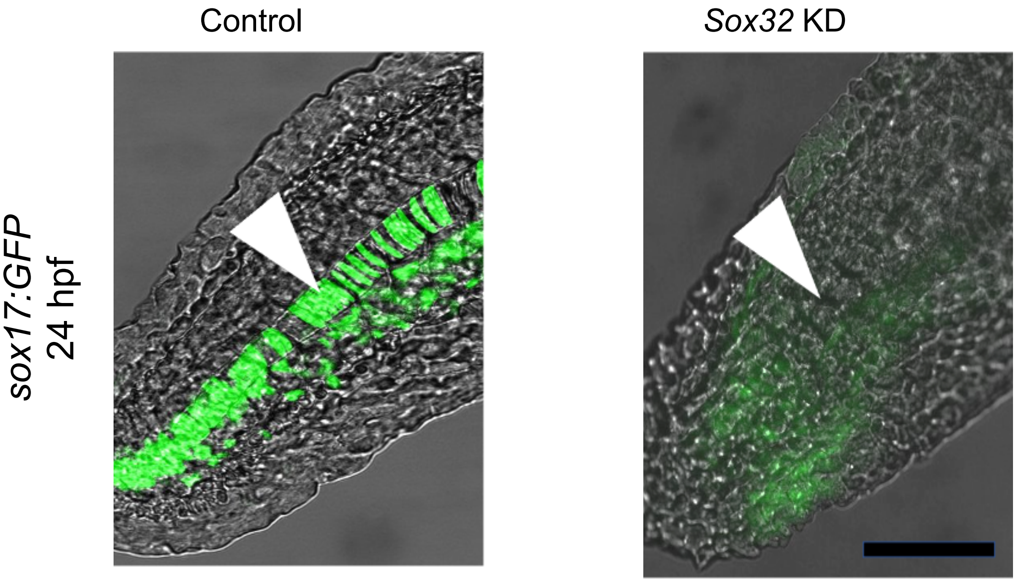

Supplemental Figure S9. GFP+ cells in the posterior notochord of sox17:GFP arise from sox32-dependent cells. *Sox17:GFP embryos injected at the one-cell stage with either control morpholino (A) or sox32 morpholino (B) and imaged at 24 hpf. The posterior notochord is shown as an overlay of fluorescence and brightfield images. Arrowhead indicates posterior notochord. Scale bar, 0.25 mm.*

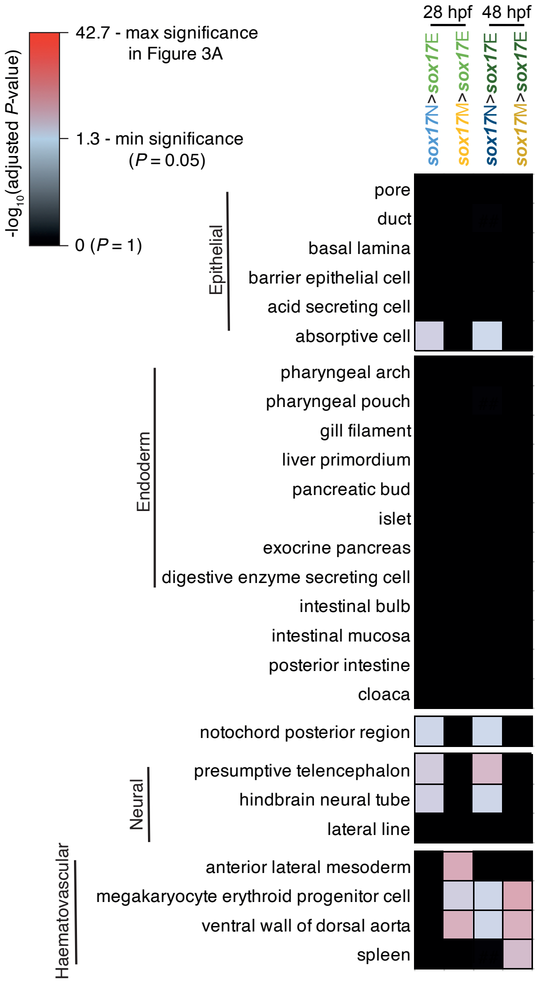

Supplemental Figure S10. Sox17N and sox17M populations are not enriched for endoderm marker promoter accessibility compared to sox17E. *Heatmap of -log$_{10}$(adjusted P-value) from fishEnrichr Anatomy GeneRIF Predicted Z-score analyses of promoters showing greater accessibility in the comparisons indicated (FDR ≤ 0.05) as annotated by ChIPseeker. Comparison with Figure 3A suggests that there is strong enrichment for endoderm populations in sox17E compared to sox17N and sox17M, but not vice versa. Furthermore, as expected, sox17N and sox17M populations show significantly greater accessibility at promoters of neural and haematovascular markers genes respectively. This strongly supports sorting of the predicted cell types into each of the sox17E, sox17N and sox17M cell populations.*

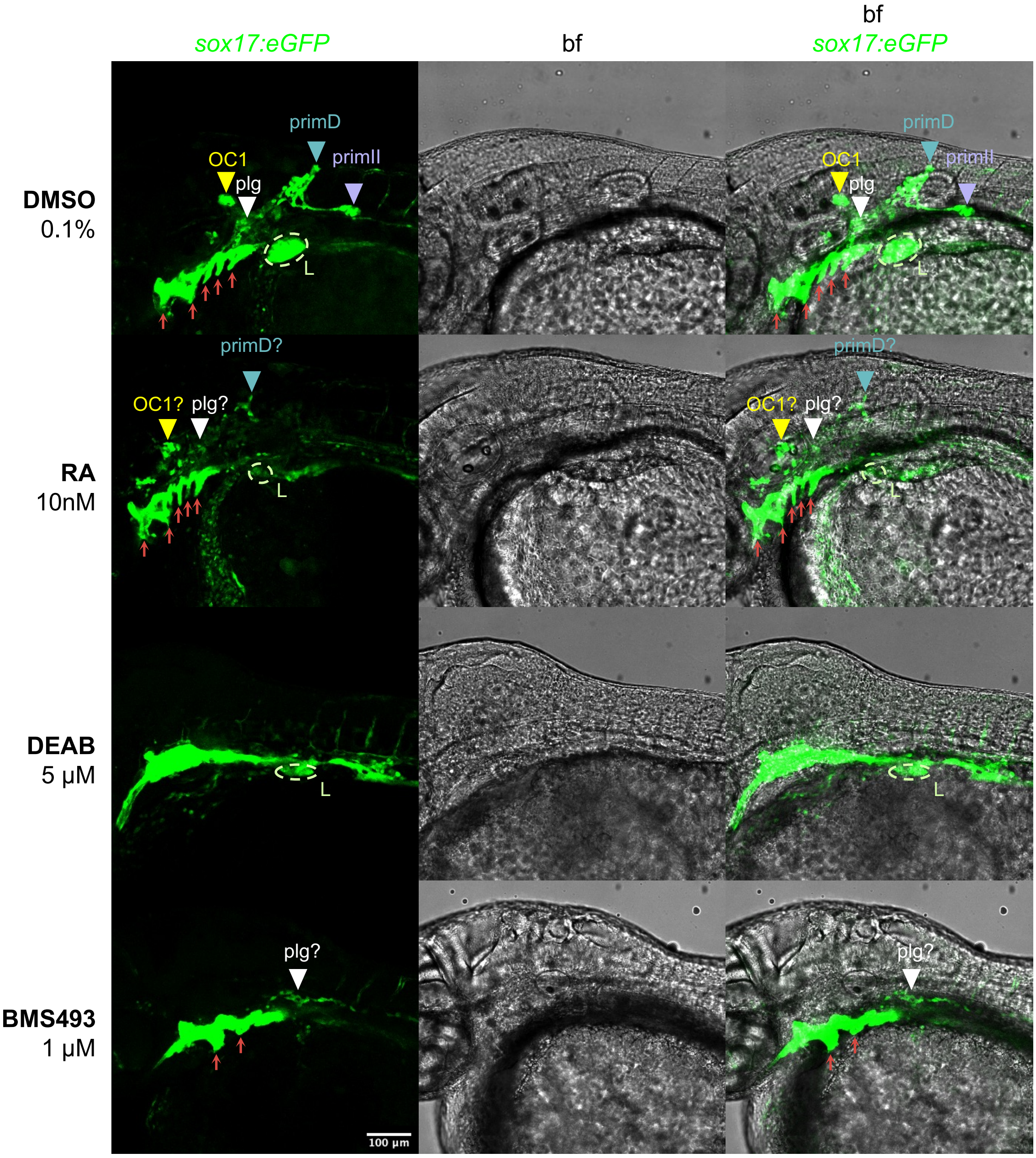

Supplemental Figure S11. sox17:GFP expression in the posterior lateral line is sensitive to RA treatment. *Z-projections of 17 slices of confocal images of 48 hpf Tg(sox17:GFP) embryos treated with DMSO (0.1%), RA (10 nM), DEAB (5 mM) or BMS493(1 mM). Laterally orientated. Images from left to right are firstly sox17:EGFP, secondly bf and thirdly overlay of bf and eGFP (green). Dorsal is top, posterior is right, ventral is bottom, anterior is left. Scale bar = 100 µm. Key estimated expression domains are marked and labelled as: OC1 = organ of Corti 1 (yellow arrow head), plg = posterior lateral line ganglion (white arrow head), primD = dorsal primordium (teal arrow head), primII = second primordium (purple arrow head), L = liver (light green dotted line), pharyngeal pouches = pp (red arrow heads). Question marks denote regions that are expected to be the expression domain based on morphology and location.*

Supplemental Table 11. Number of H3K27ac peaks called in each cell population drops with more stringent q-values

Cell population	Number of peaks *q*-value <0.05	*q*-value <10^{-8}
AFG	153,956	69,853
PFG	150,374	72,194
MHG	129,060	56,159

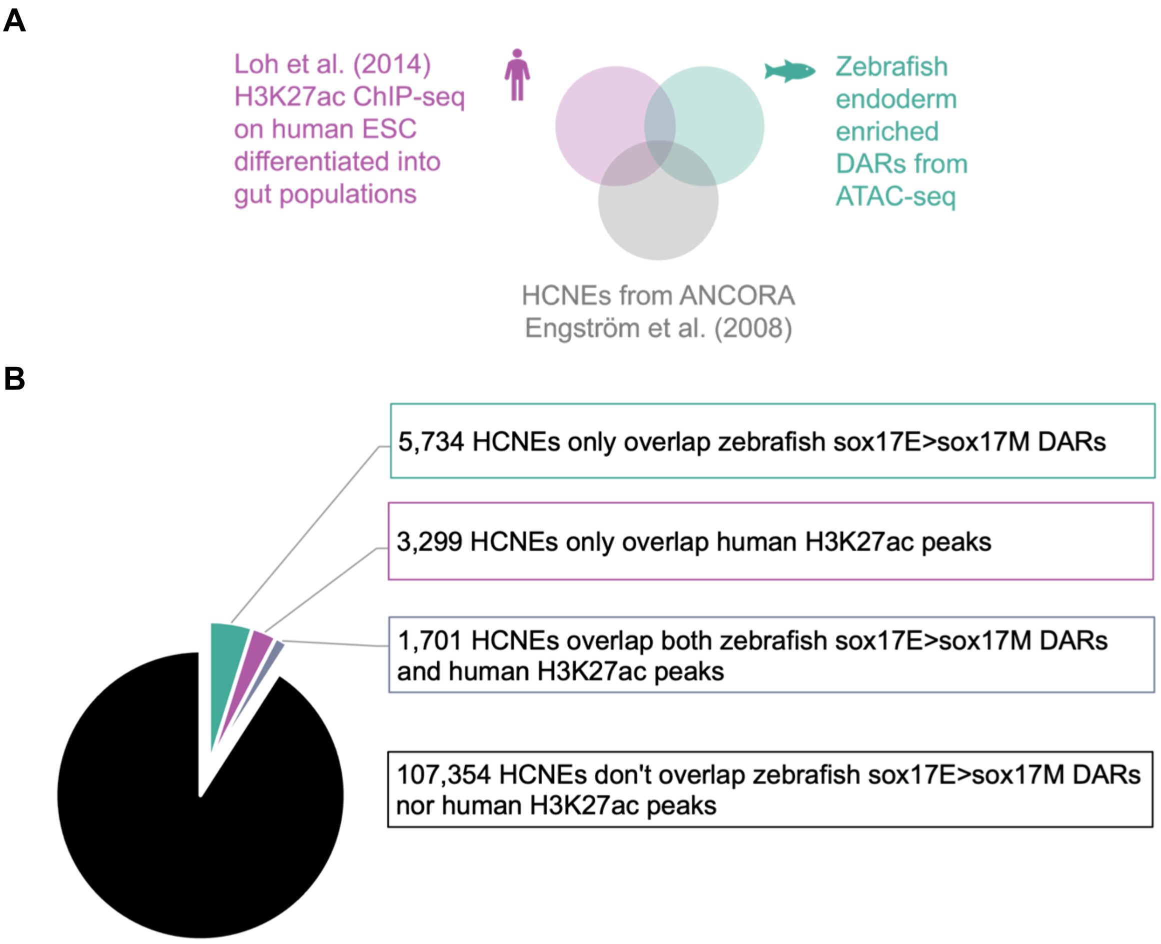

Supplemental Figure S12. Human endoderm H3K27ac peaks and/or zebrafish sox17E>sox17M DARs overlap with 9% of zebrafish/human HCNEs. *(A) Schematic of the overlap of HCNEs from ANCORA with the functional genomics data of H3K27ac peaks in hESCs differentiated into anterior posterior patterned endodermal cells from Loh et al. (2014) and the endoderm enriched sox17E>sox17M DARs from 28 and 48 hpf zebrafish embryos collected by us using ATAC-seq. (B) Pie chart showing the number of HCNEs overlapping the two data sets aforementioned.*

Supplemental Table 13. Number of HCNEs overlapping zebrafish sox17E>sox17M DARs and H3K27ac peaks in anterior-posterior patterned endoderm cell populations derived from hESCs

		What HCNEs overlap					Number of HCNEs		
**Name of category**	**Name of overlap**	**Zebrafish sox17E>sox17M DARs**		**Human H3K27ac peaks**			**in overlap**	**in total category**	
		**28 hpf**	**48 hpf**	**AFG**	**PFG**	**MHG**			
**Only overlap sox17E>sox17M DARs**	28						2,392		
	48						1,886		
	28 48						1,456		5,734
**Overlap both sox17E>sox17M DARs and H3K27ac peaks**	28 AFG						104		
	28 AFG PFG						169		
	28 PFG						245		
	28 PFG MHG						38		
	28 MHG						77		
	28AFG MHG						29		
	28 AFG PFG MHG						116	778	
	48 AFG						54		
	48 AFG PFG						35		
	48 PFG						95		
	48 PFG MHG						11		
	48 MHG						70		
	48 AFG MHG						17		
	48 AFG PFG MHG						61	343	
	28 48 AFG						80		
	28 48 AFG PFG						110		
	28 48 PFG						165		
	28 48 PFG MHG						25		
	28 48 MHG						75		
	28 48 AFG MHG						33		
	28 48 AFG PFG MHG						92	580	1,701
**Only overlap H3K27ac peaks**	AFG						684		
	AFG PFG						294		
	PFG						913		
	PFG MHG						97		
	MHG						484		
	AFG MHG						173		
	AFG PFG MHG						654		3,299
**Don't overlap sox17E>sox17M DARs nor H3K27ac peaks**							107,354		107,354
		5,206	4,265	2,705	3,120	2,052			**118,088**

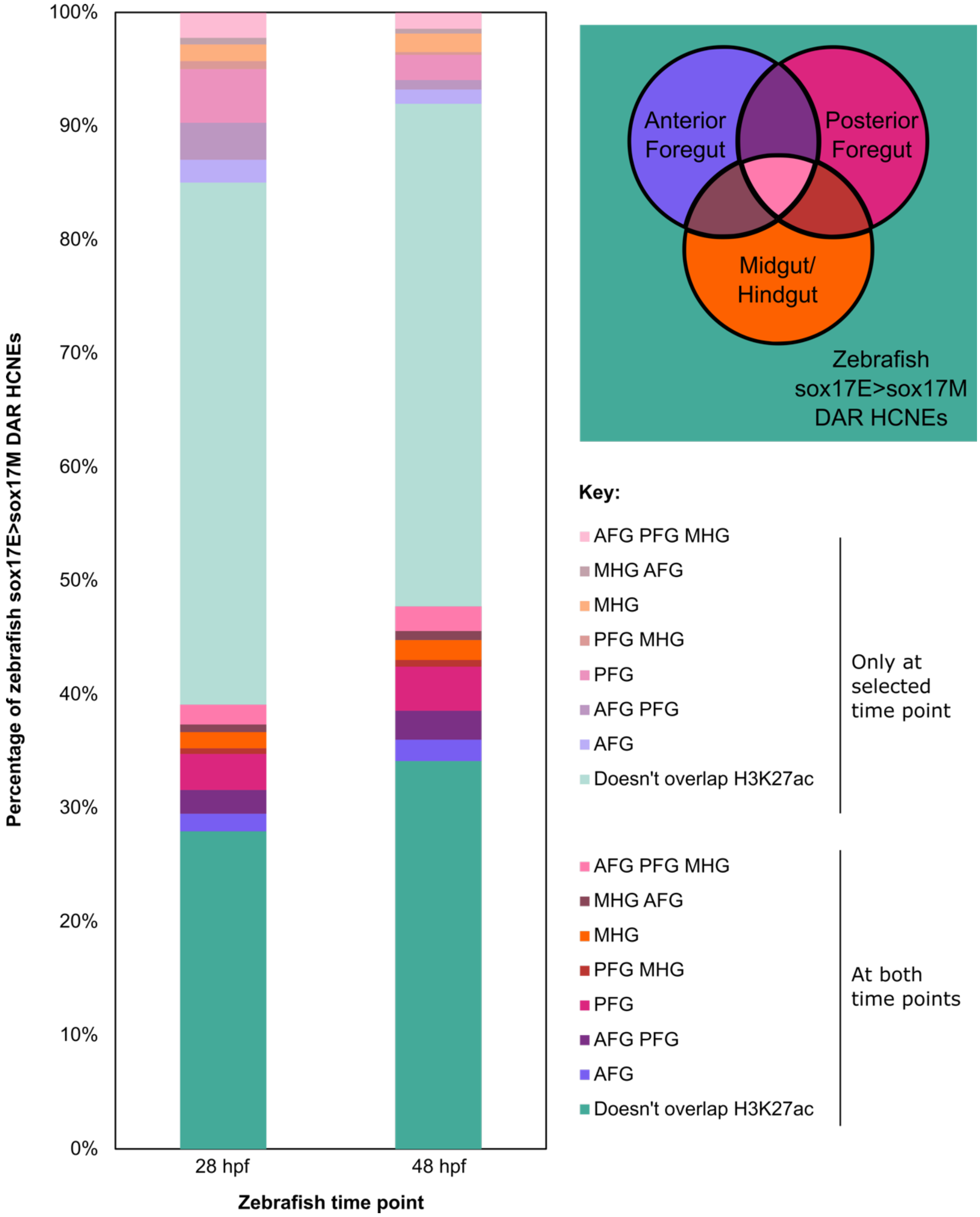

Supplemental Figure S13. Overlap of HCNEs with sox17E>sox17M DARs and H3K27ac. *Bar chart to show what percentage of HCNEs that overlap zebrafish sox17E>sox17M DARs at 28 or 48 hpf also overlap human H3K27ac in anterior-posterior patterned endodermal cells and/or zebrafish DARs at the other time point. Venn diagram on the right shows colours used for bar chart with reference to which human endodermal cell population the H3K27ac signal comes from that overlaps the zebrafish sox17E>sox17M DAR HCNEs. Zebrafish sox17E>sox17M DAR HCNEs that do not overlap human endodermal cell population H3K27ac signal are shown in teal.*

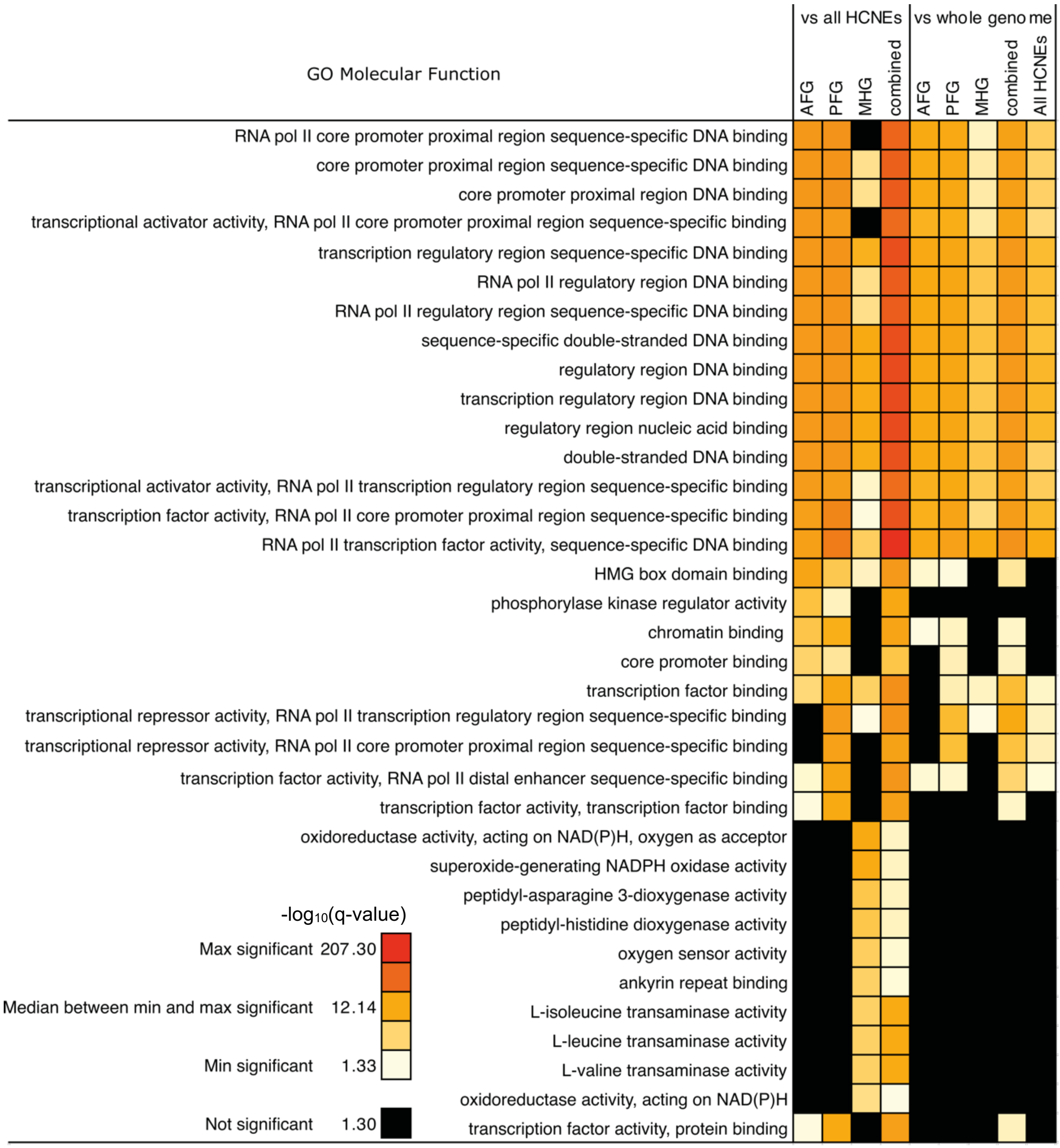

Supplemental Figure S14. DNA-binding terms are found in top GO molecular function terms enriched in genes near endodermal HCNEs. *HCNEs overlapping sox17E>sox17M zebrafish DARs and H3K27ac peaks in anterior-posterior patterned endoderm cell populations derived from hESCs was used as GREAT input. Terms ranked based on highest -log_{10}(HyperFdrQ). Top 20 terms enriched vs all HCNEs for AFG only, PFG only, MHG only and AFG/PFG/MHG combined were selected and are displayed in that order, with replicates only shown once. Heatmap based on the -log_{10}(HyperFdrQ). Not significant values are shown in black and have a HyperFdrQ<=0.05 and -log_{10}(HyperFdrQ) <= 1.30. Significant values are coloured on a scale from minimum significance -log_{10}(HyperFdrQ) in cream, to maximum significance -log_{10}(HyperFdrQ) in red. The median -log_{10}(HyperFdrQ) value between minimum and maximum significant -log_{10}(HyperFdrQ) is orange. Discrete colour coded values in between indicate 25th, 50th and 75th percentile -log_{10}(HyperFdrQ) for significant terms.*

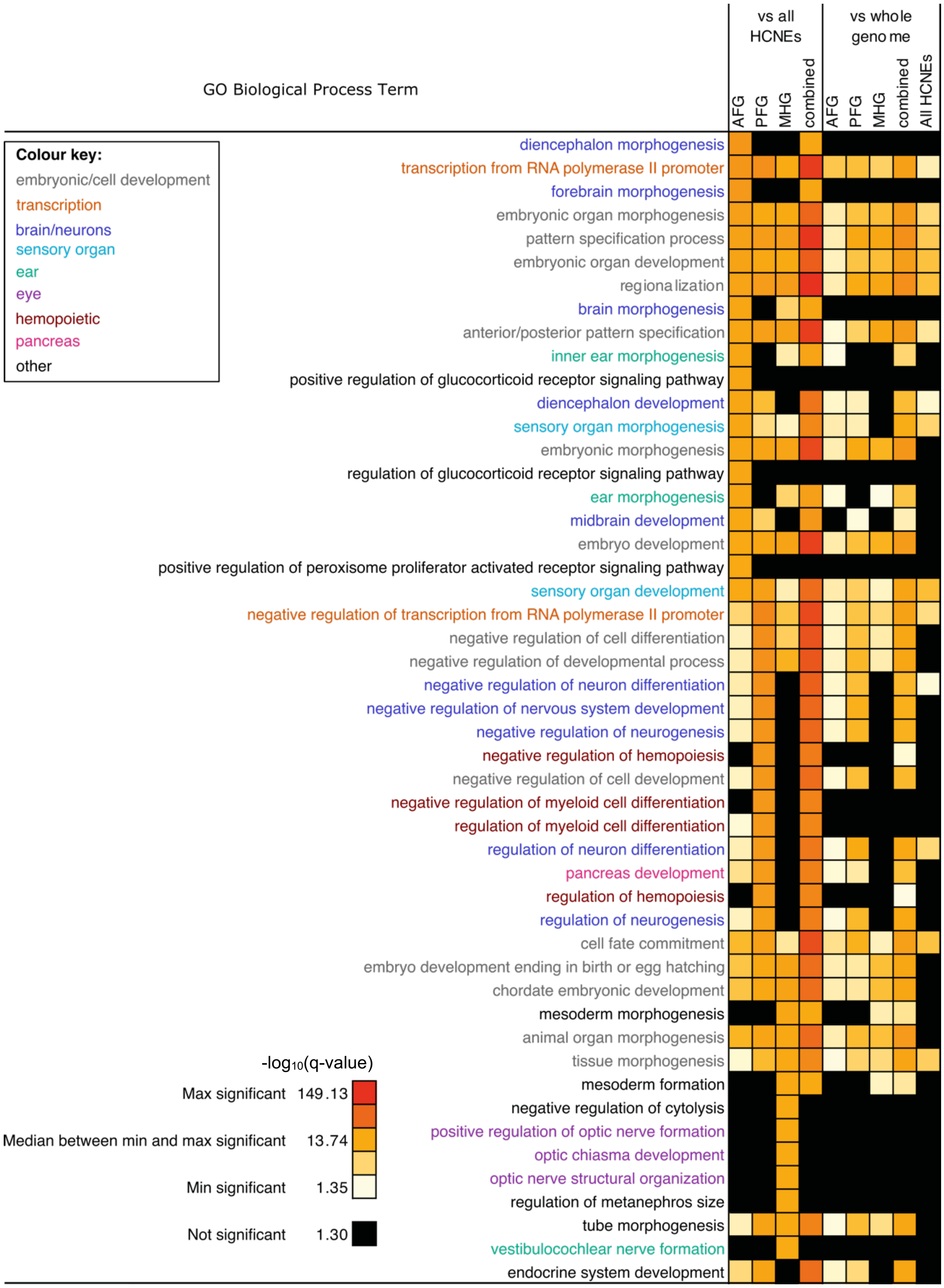

Supplemental Figure S15. There is enrichment for embryonic development and transcription regulation in the top GO biological process terms enriched in genes near endodermal HCNEs *Processed as in (Supplemental Figure S14). Related terms have been coloured as shown in the colour key.*

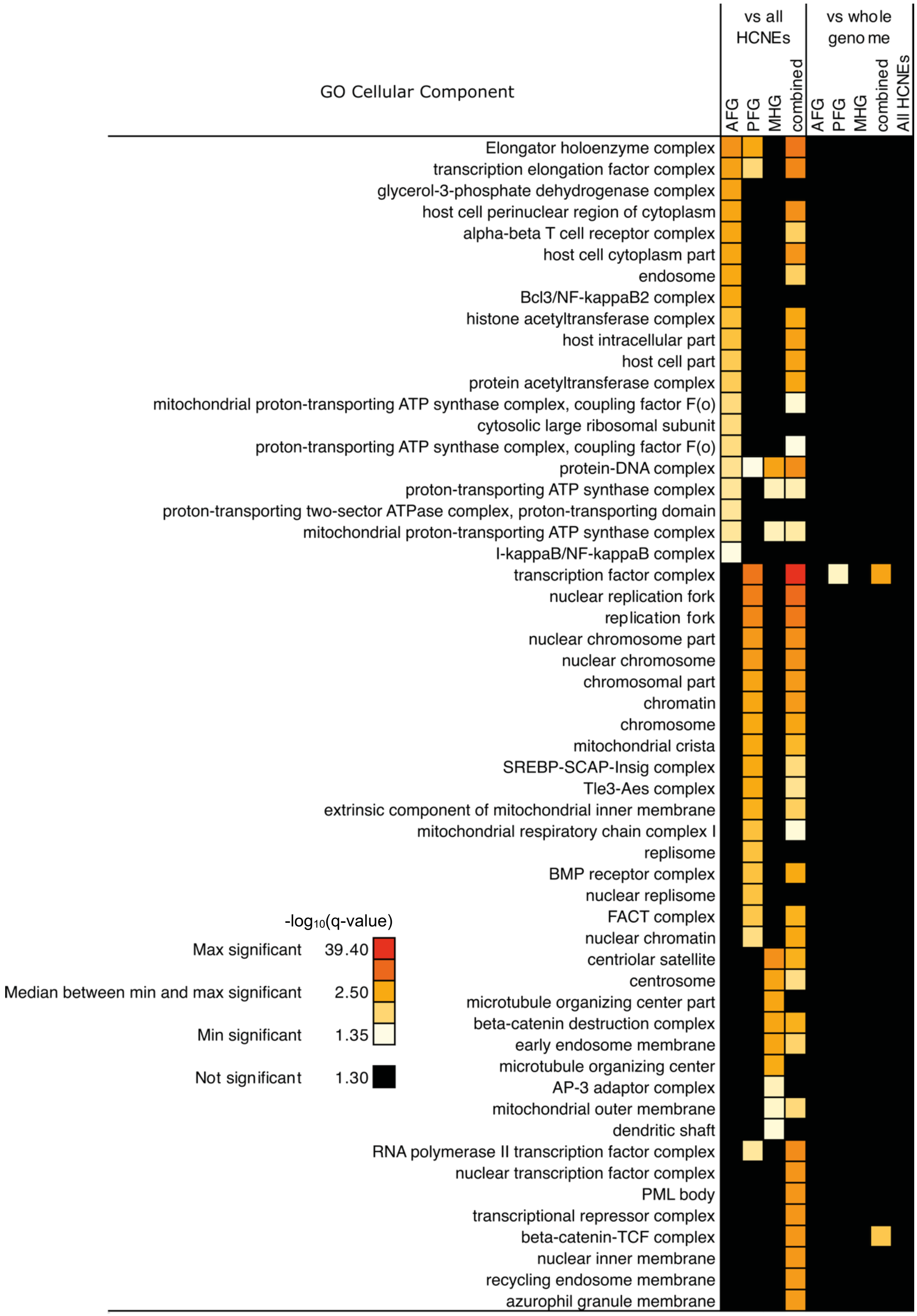

Supplemental Figure S16. There is enrichment for transcription terms in the top GO cellular component terms enriched in genes near endodermal HCNEs. *Processed as in (Supplemental Figure S14).*

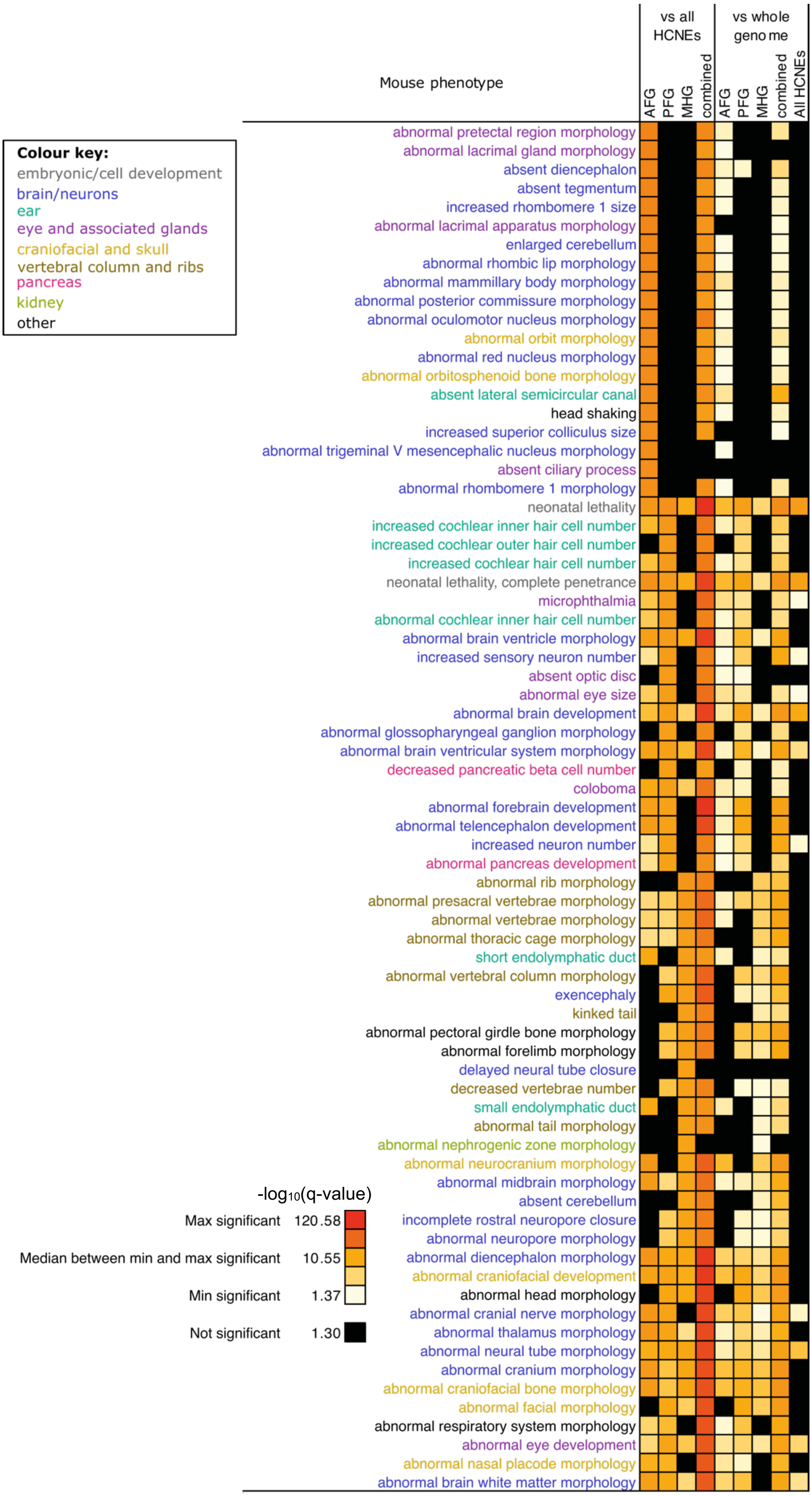

Supplemental Figure S17. A range of mouse phenotypes are enriched in endodermal HCNE associated genes. *Processed as in (Supplemental Figure S14). Related terms have been coloured as shown in the colour key.*

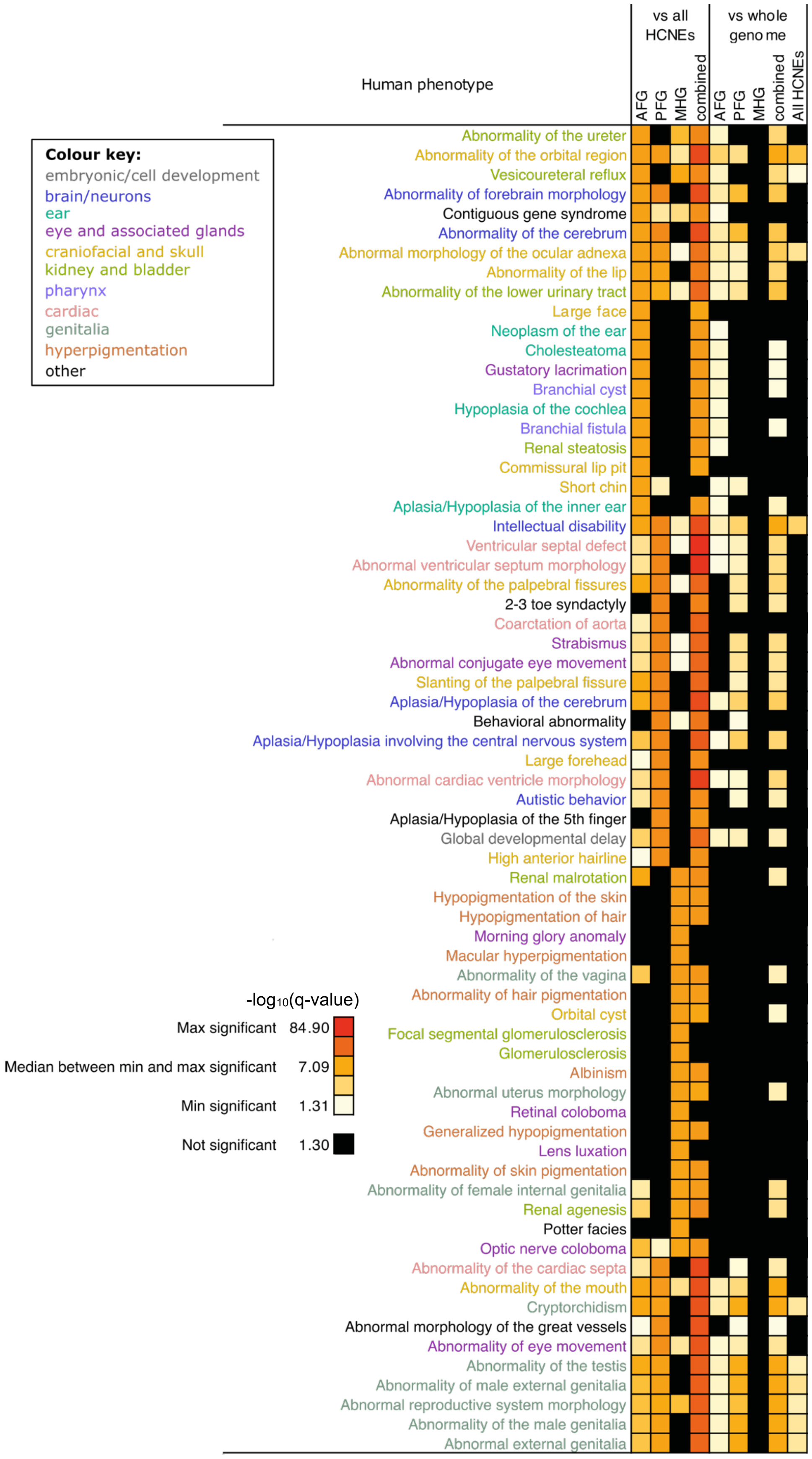

Supplemental Figure S18. There is enrichment for a variety of phenotypes in the top human phenotype terms enriched in genes near endodermal HCNEs. *Processed as in Supplemental Figure S14. Related terms have been coloured as shown in the colour key.*

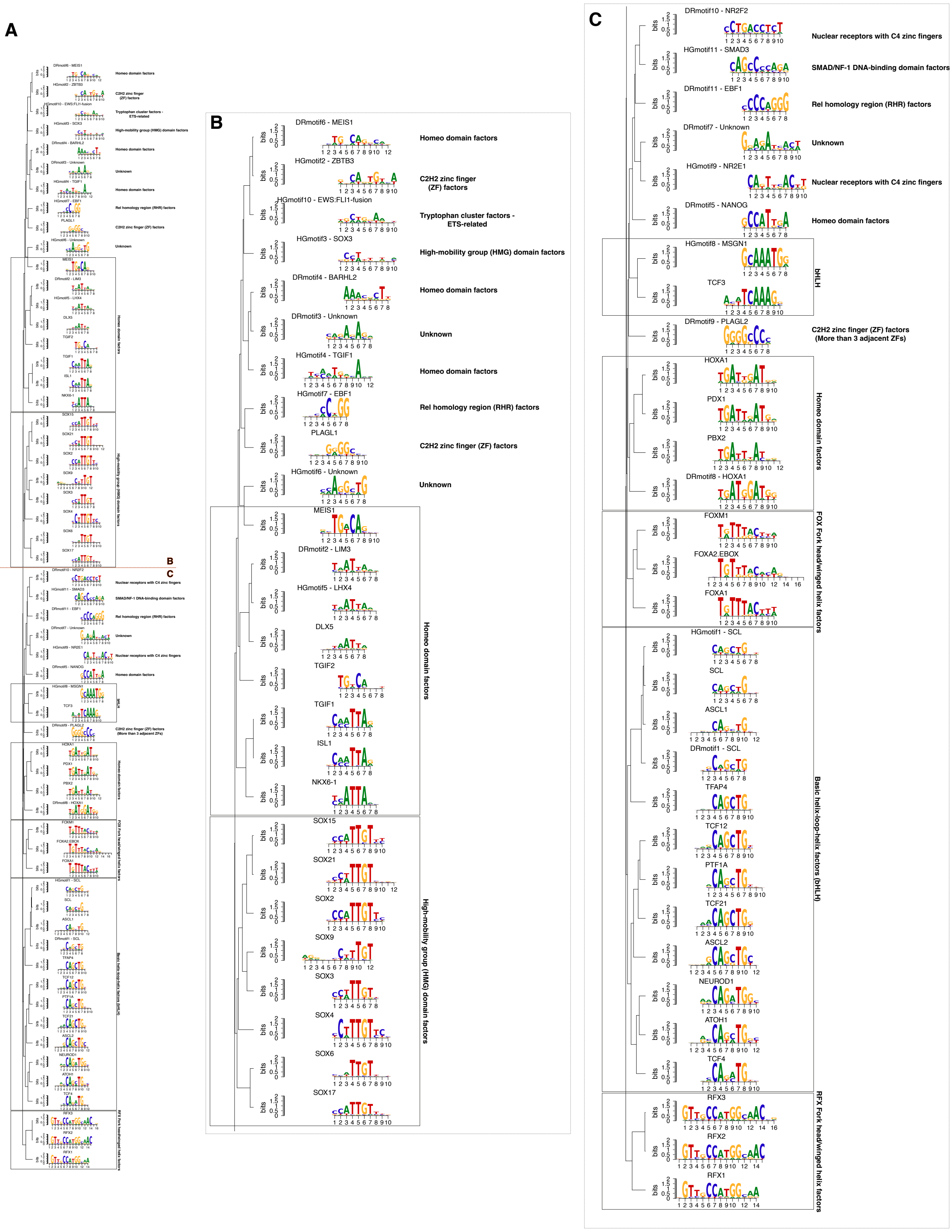

Supplemental Figure S19. Clustering of known and de novo motifs corresponding to Figure 5. *Hierarchical clustering of all motifs (panel A) using motifStack (Ou et al. 2018). Motifs above the brown line are depicted enlarged in panel B, and those below the line in panel C. Subsets of motifs are boxed and annotated according to the transcription factor families they belong to. Note that some motif subsets are sufficiently similar that they can likely be bound by multiple factors.*

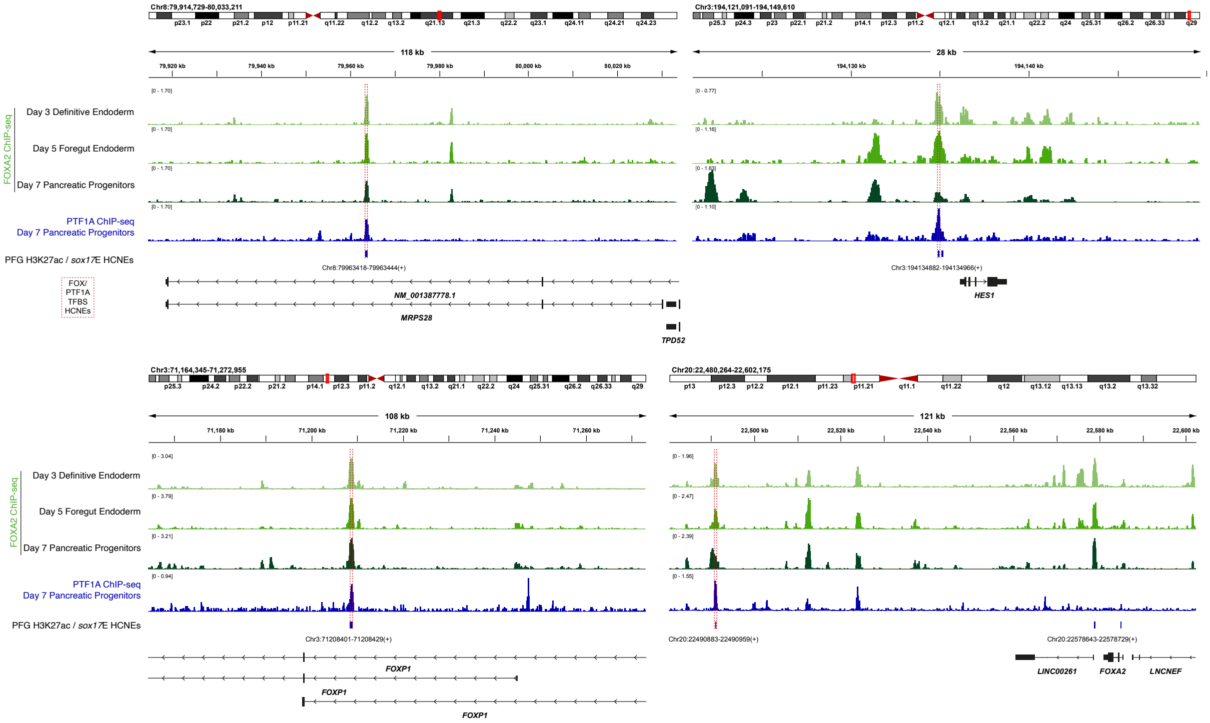

Supplemental Figure S20. FOXA2 binds at HCNEs during hESC to pancreatic endoderm differentiation, followed by subsequent PTF1A binding. *Four example loci identified through intersecting published FOXA2 and PTF1A ChIP-seq data (Lee et al. 2019; Miguel-Escalada et al. 2022) with PFG HCNEs identified in the present study. Stages of differentiation and TF are as indicated on the left. Hg38 genomic coordinates are provided. ChIP-seq peaks with submits at PFG HCNEs are in red boxes. Track scales in CPM are indicated. Note that boxed HCNEs proximal to the FOXA2 and HES1 genes are also amongst examples highlighted in Figure 4.*

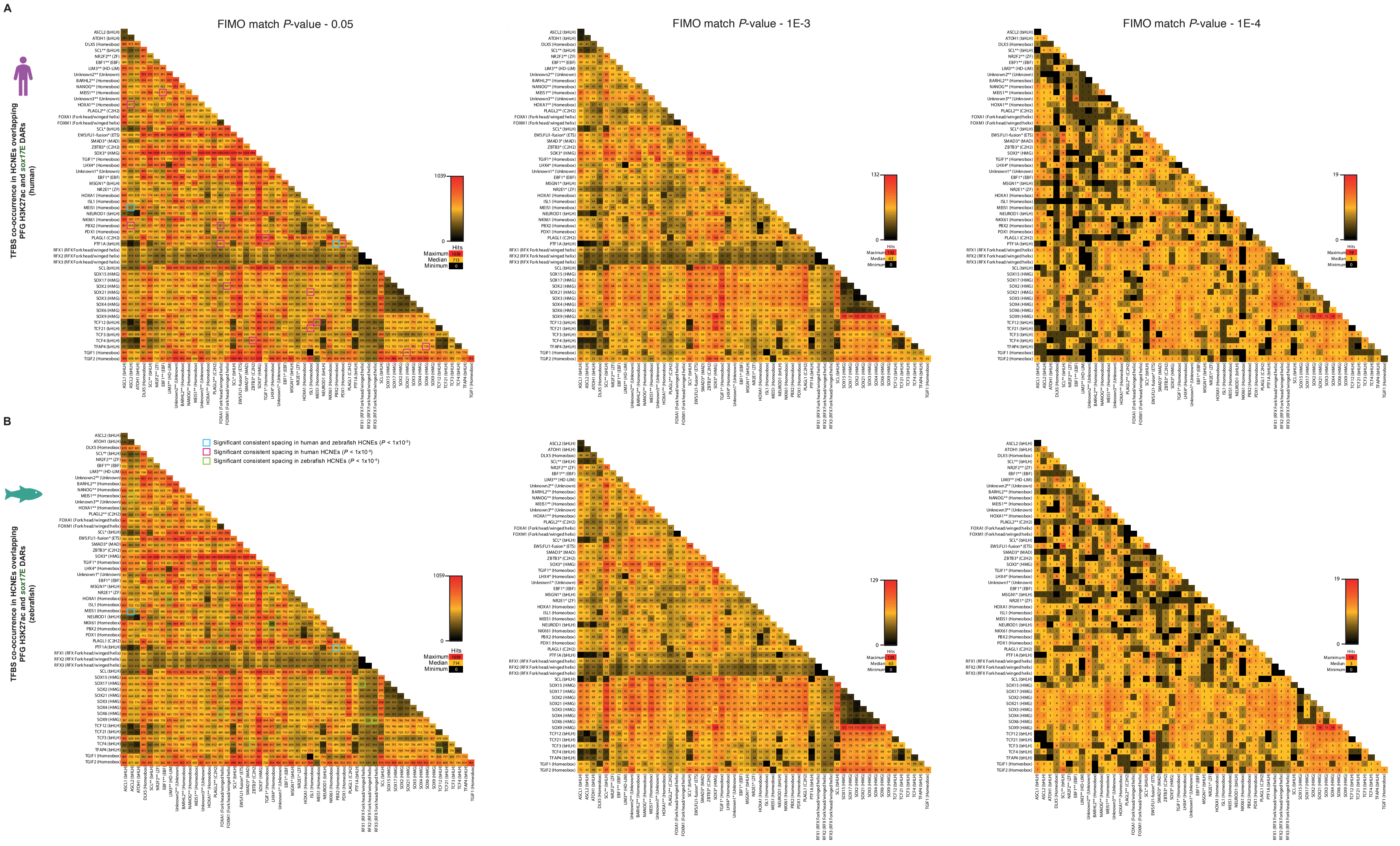

Supplemental Figure S21. Human and zebrafish endoderm HCNEs show consistent coincidence of transcription factor binding sites (TFBSs) but with limited grammatical consistency either within or between species. *(A) Heatmap matrix indicating the number of human HCNEs overlapping both human PFG H3K27ac peaks and sox17E>sox17M DARs at 28 and/or 48 hpf containing a significant match for motif pairs as indicated. Intersecting cells corresponding to TFBSs on the horizontal and vertical axes indicate the number of HNCEs in which the two TFBSs co-occur. Successive matrix heatmaps indicate TFBS identification using FIMO at the P-value cutoffs indicated above. Continuous scale key insets indicate percentile incidence of cooccurrence. (B) As panel A but for zebrafish HCNE sequences. Co-occurring motifs showing significantly consistent spacing (SpaMo P-value < 1 x 10^{-5}) are outlined and colour-coded in the lefthand heatmaps to represent whether significantly consistent spacing is found in human or zebrafish HCNEs or both. Co-occurring motifs showing consistent spacing are further depicted in Figure 6. * De novo motif from human HCNEs; ** de novo motif from zebrafish HCNEs. Note that for TFBSs to be called as co -occurring they must be at distinct coordinates. Highly similar motifs therefore do not tend to be identified as co-occurring. See the cluster of SOX factor TFBSs as an illustration of this.*

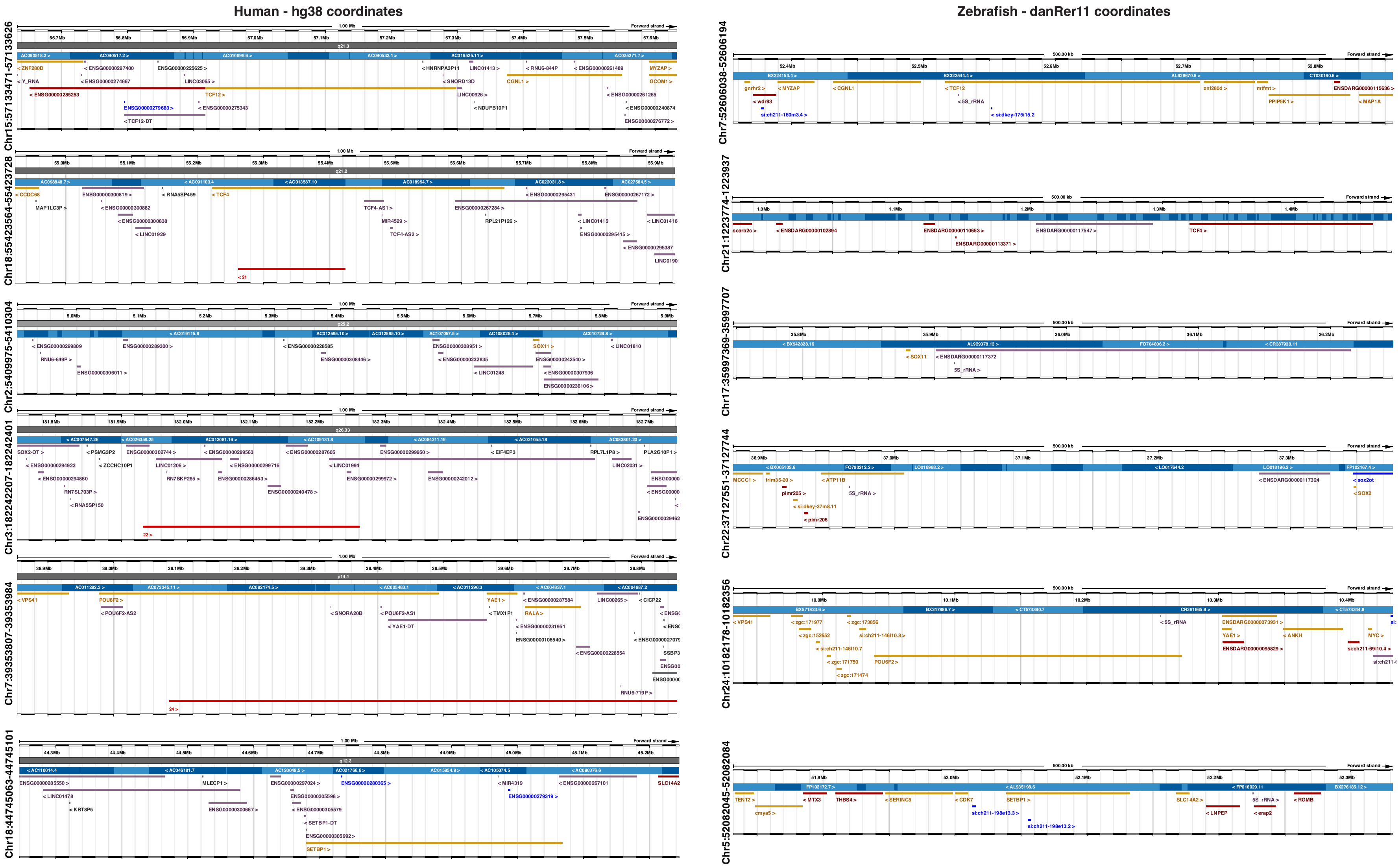

Supplemental Figure S22. Location of orthologous human-zebrafish HCNEs exhibiting consistent 12 bp spacing of bHLH and homeodomain TFBSs indicated in Figure 6. *HCNE locations are in the centre of each window. 1 Mb of human genome and 500 kb of zebrafish genome are shown, reflecting the relative sizes and compactness of each genome. Genes depicted are as in Ensembl version 114. Equivalent human and zebrafish genomic locations are depicted side-by-side, and in the order represented in Figure 6C.*

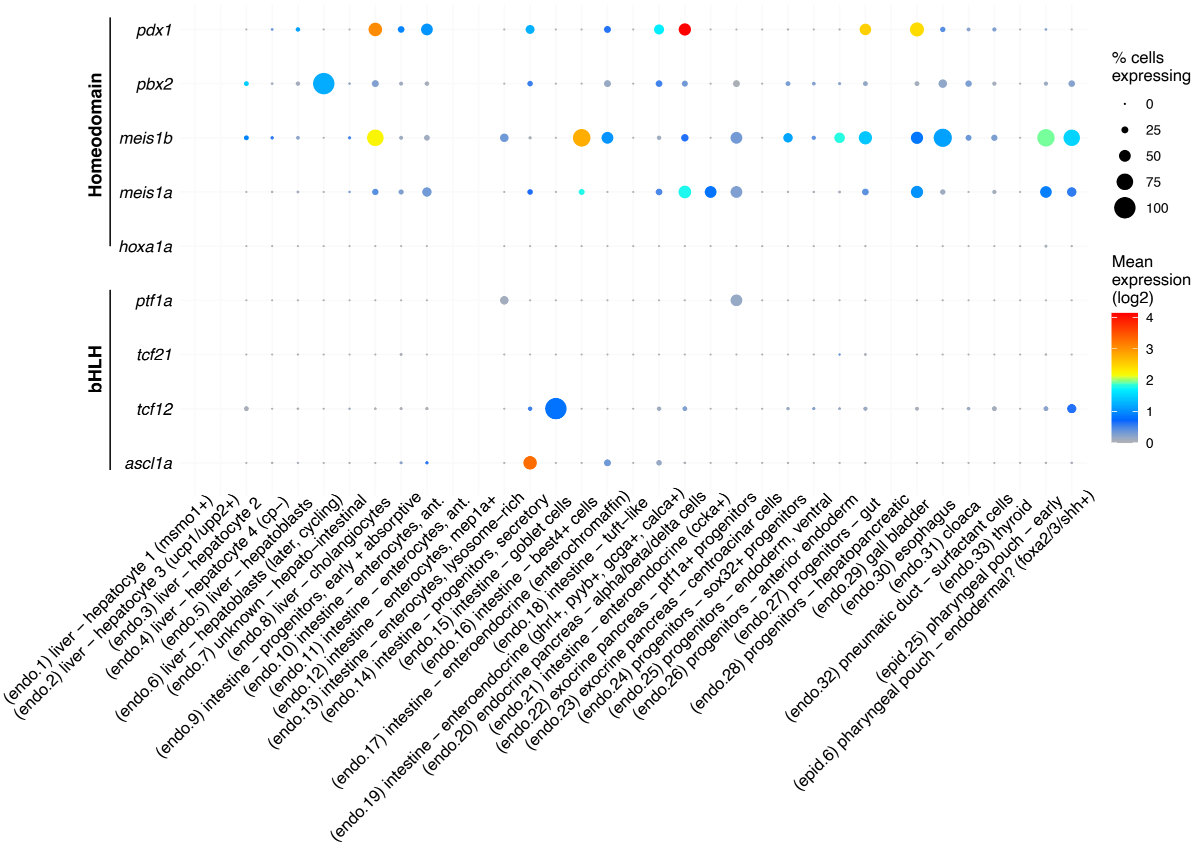

Supplemental Figure S23. Co-expression in developing zebrafish endoderm of homeodomain-bHLH TF pairs depicted in Figure 6. *The dotplot was produced for all endoderm cell identities defined in the Daniocell database using the DaniocellDesktop application (Farrell et al. 2018; Sur et al. 2023).*

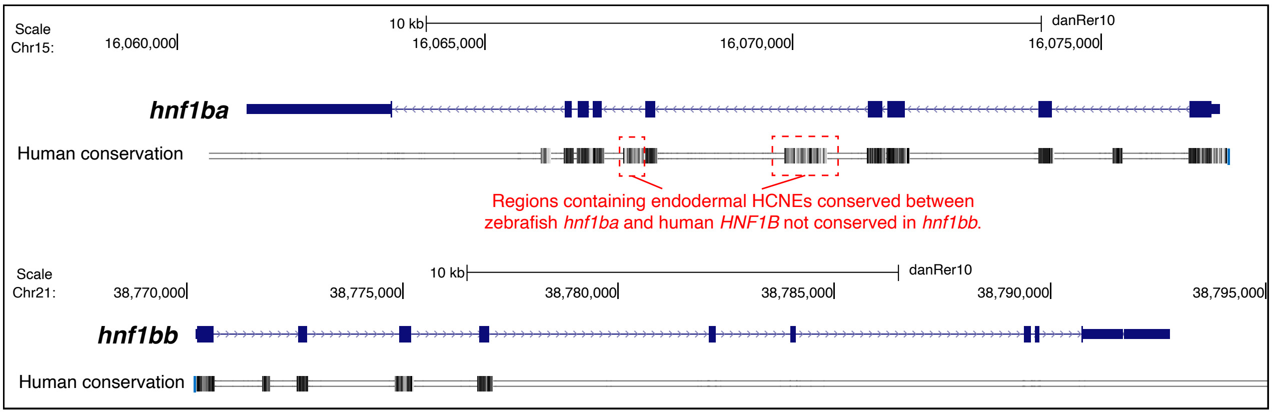

Supplemental Figure S24. HCNEs in introns 4 and 5 of zebrafish hnf1ba and human HNF1B are not conserved in zebrafish hnf1bb. *Multiz alignments of zebrafish and human genomes are shown. Conserved regions containing endodermal HCNEs identified in our analyses are highlighted in red boxes.*

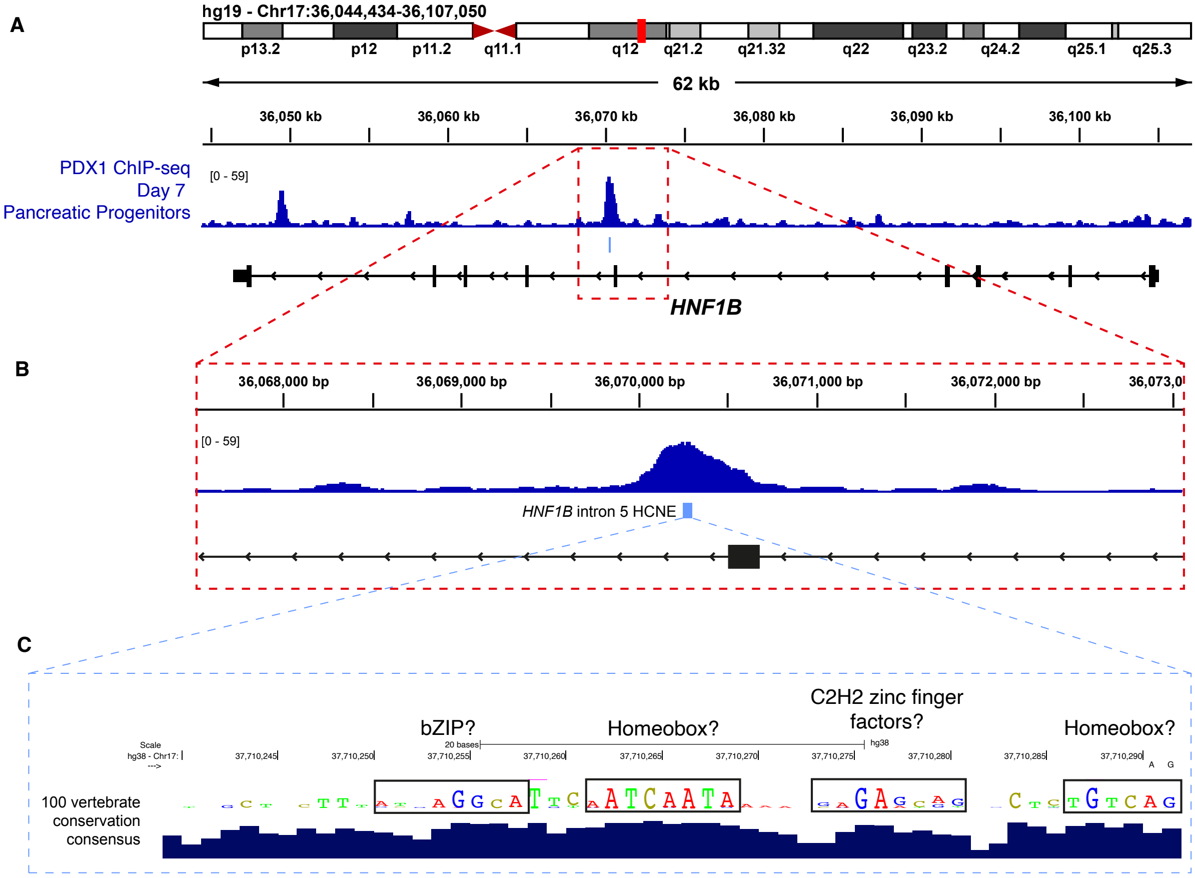

Supplemental Figure S25. PDX1 binds the intron 5 *HNF1B* HCNE in human pancreatic progenitors. *(A) ChIP-seq data for homeodomain transcription factor PDX1 from day 7 pancreatic progenitor cells differentiated from hESCs (Lee et al. 2019). (B) Zoomed view as indicated by the red box. The peak summit appears to coincide with the intron 5 HCNE. (C) Consensus sequence of the intron 5 HCNE across alignments of 100 vertebrate species from UCSC genome browser. Annotated are potential classes of transcription factor binding sites predicted using Tomtom (Gupta et al. 2007).*

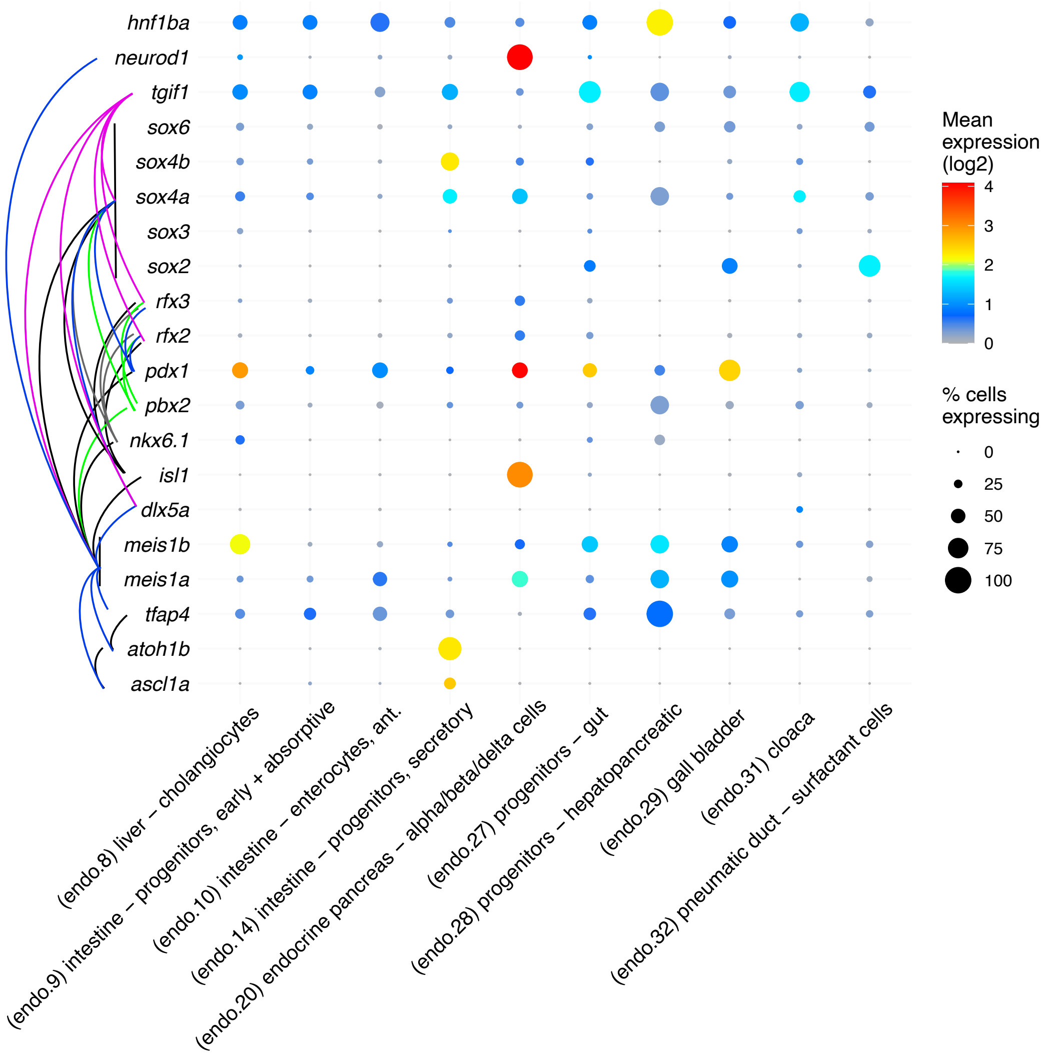

Supplemental Figure S26. Hnf1ba is co-expressed with TF genes whose TFBSs co-occur in intron 4 HCNEs. *Dotplot indicating expression of hnf1ba in hnf1ba+ endoderm populations and TF genes with TFBSs identified as co-occurring in human and zebrafish intron 4 HCNEs at FIMO P-value < 1E-3 (Supplemental Fig. 21). The dotplot was produced using the DaniocellDesktop application (Farrell et al. 2018; Sur et al. 2023). Sets of TFBSs showing pairwise co-occurrence are linked by lines coloured to allow individual lines to be more easily distinguished.*

Supplemental Table 14. Coordinates of putative enhancers and HCNEs of hnf1ba studied

**Enhancer name**	**Chromosome coordinates in genome versions**			
	**danRer7**	**danRer10**	**danRer11**	**hg19**
I5Enh +9Kb	Chr15:15021553-15022991	Chr15:16066516-16067954	Chr15:16002538-16003976	Chr17:36061008-36070674
I5zHCNE	Chr15:15022306-15022646	Chr15:16067269-16067609	Chr15:16003291-16003631	Chr17:36070162-36070510
I5hHCNE	Chr15:15022306-15022725	Chr15:16067269-16067688	Chr15:16003291-16003710	Chr17:36070108-36070589
I4 Enh +8Kb	Chr15:15023504-15024371	Chr15:16068467-16069334	Chr15:16004489-16005356	Does not map
I4 Enh +6Kb	Chr15:15025324-15026283	Chr15:16070287-16071246	Chr15:16006309-16007268	Chr17:36087496-36091612
I4 Enh +6-8Kb	Chr15:15023504-15026283	Chr15:16068467-16071246	Chr15:16004489-16007268	Chr17:36087174-36091612
I4zHCNE	Chr15:15024917-15025560	Chr15:16069880-16070523	Chr15:16005902-16006545	Chr17:36087165-36087758
I4hHCNE	Chr15:15024917-15025560	Chr15:16069880-16070523	Chr15:16005902-16006545	Chr17:36086990-36087861
Enh -3Kb	Chr15:15034801-15035669	Chr15:16079764-16080632	Chr15:16015786-16016654	Does not map

Supplemental Table 15. Table showing where enhancers drove reporter expression in developing zebrafish at 48 hpf

For each construct injected the number of embryos (first column) and percentage of embryos screened (second column) that show the observed expression are shown. Intensity of magenta represents percentage of embryos screened showing expression. N refers to biological replicates of experiment, n refers to number of embryos screened. Not scorable embryos are due to there being no expression observed and/or masking of signal due to YSL autofluorescence.

**Expression observed**	**Enhancer construct injected**															
	**i5**				**i4**											
	**zHCNE**		**hHCNE**		**Enh +6-8Kb**		**Enh +8Kb**		**Enh +6Kb**		**zHCNE**		**hHCNE**		**Enh -3Kb**	
Hindbrain	2	28.6	8	88.9	4	28.6	0	0.0	3	42.9	28	62.2	14	87.5	0	0.0
Forebrain	0	0.0	4	44.4	0	0.0	1	14.3	0	0.0	1	2.2	0	0.0	0	0.0
Mouth/jaw	3	42.9	0	0.0	0	0.0	0	0.0	0	0.0	0	0.0	0	0.0	4	26.7
Neural tube	0	0.0	0	0.0	7	50.0	6	85.7	0	0.0	3	6.7	1	6.3	0	0.0
Floor plate	0	0.0	0	0.0	5	35.7	0	0.0	0	0.0	1	2.2	0	0.0	0	0.0
Pericardium and/or heart	4	57.1	3	33.3	0	0.0	0	0.0	0	0.0	0	0.0	0	0.0	0	0.0
Blood	0	0.0	4	44.4	0	0.0	1	14.3	0	0.0	0	0.0	0	0.0	0	0.0
Tail muscle cells	0	0.0	2	22.2	0	0.0	4	57.1	0	0.0	3	6.7	1	6.3	0	0.0
Not scorable	0	0.0	1	11.1	0	0.0	0	0.0	3	42.9	17	37.8	1	6.3	0	0.0
Number of embryos screened (n)	7	100.0	9	100.0	14	100.0	7	100.0	7	100.0	45	100.0	16	100.0	15	100.0

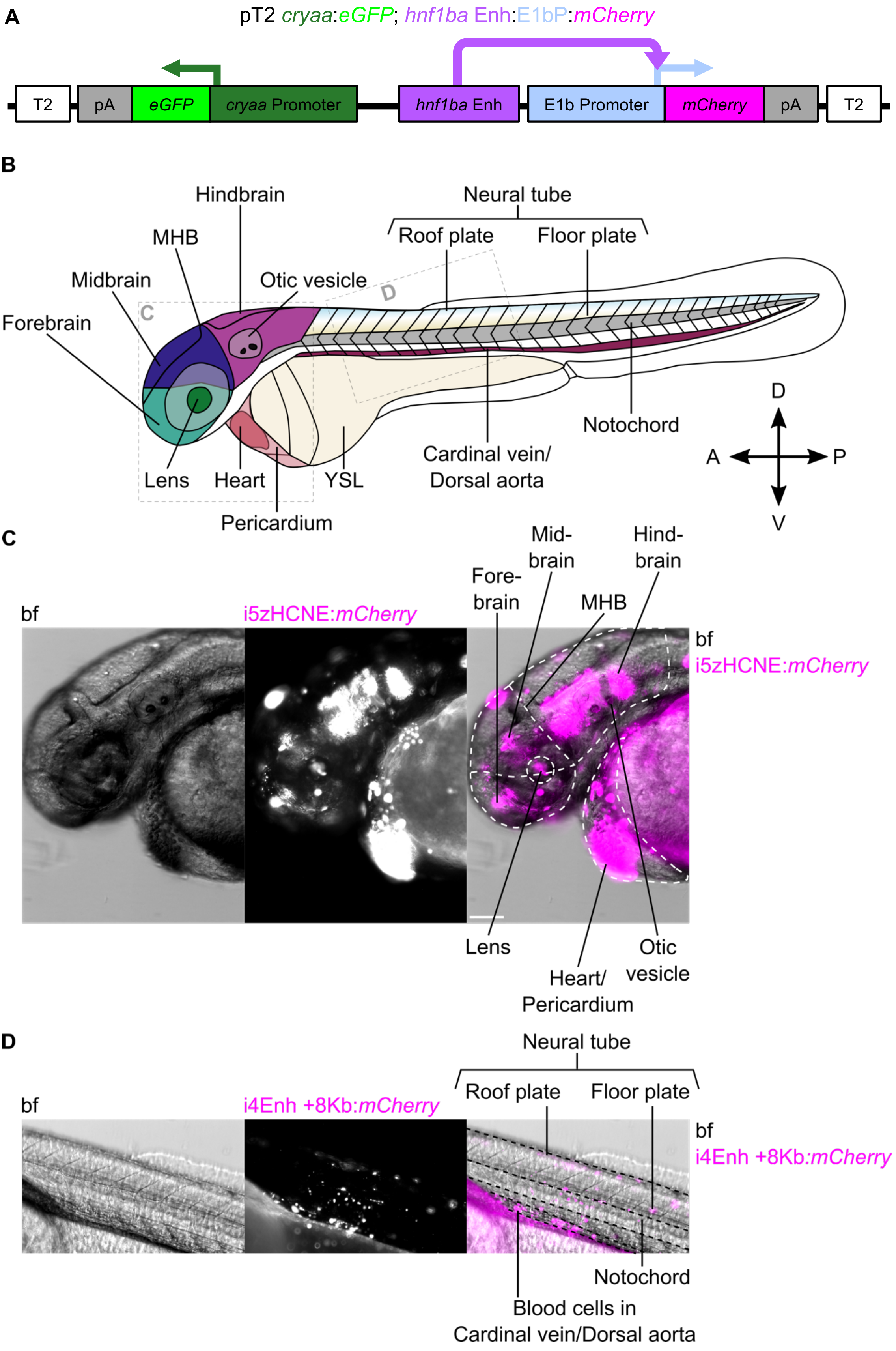

Supplemental Figure S27. Putative hnf1ba enhancers show reporter expression in multiple cell types including in the brain and neural tube

(A) Schematic of reporter construct injected alongside Tol2 mRNA for reporter assay. Green arrow indicates cryaa driving eGFP expression. Purple arrow indicates putative enhancer enhancing mCherry

expression. Blue arrow indicates E1b promoter driving mCherry expression. (B) Schematic of expression domains seen in zebrafish at ~48 hpf. Gray boxes with letters denote the approximate location of the images shown in C and D. For the anatomical direction: D = dorsal, P = posterior, V= ventral, A = anterior. MHB = midbrain hindbrain boundary. YSL = yolk syncytial layer. (C-D) Images from 48 hpf embryos injected with reporter constructs to demonstrate some of the expression patterns seen. Left is bf, middle is reporter with basal promoter E1b driving mCherry expression, and right is overlay of bf and mCherry (magenta). Lateral views. Scale bar = 100mm. Contrast settings are the same for both C and D. Dashed lines show approximate boundaries for anatomical structures labelled. (C) i5zHCNE driving expression in forebrain, midbrain, hindbrain, and heart/pericardium. (D) i4Enh +8Kb driving expression in the roof plate and floor plate of the neural tube, as well as the blood cells in the cardinal vein/dorsal aorta.

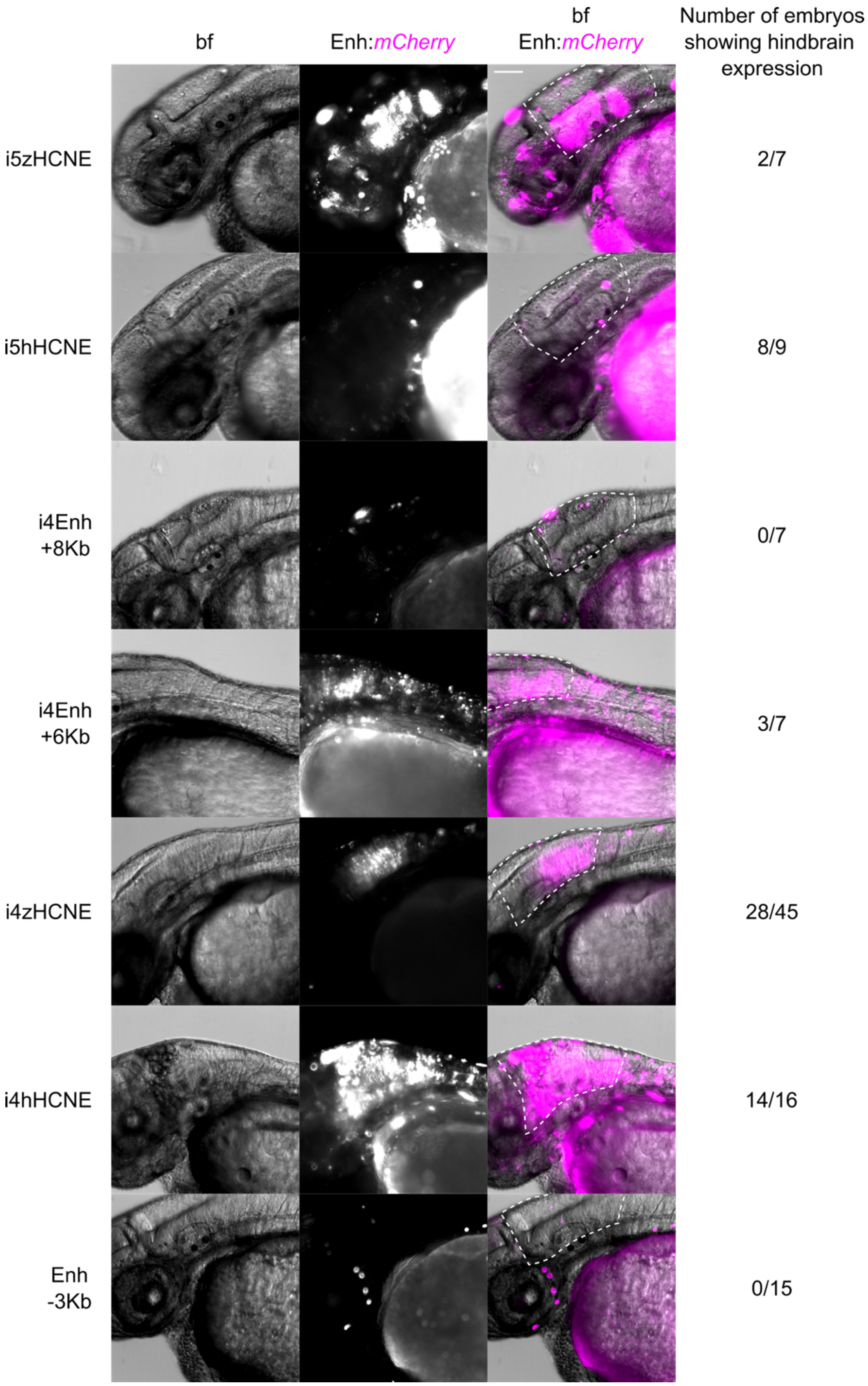

Supplemental Figure S28. Consistent expression in the hindbrain driven by hnf1ba putative enhancers. *Images of heads of 48 hpf embryos injected with reporter construct. Lateral views. All are dorsal up, and anterior on the left (i4zHCNE has been flipped horizontally for ease of comparison). Left is bf, middle is enhancer with basal promoter E1b driving mCherry expression, and right is overlay of bf and mCherry (magenta). White dashed line marks hindbrain. Scale bar = 100mm. Contrast settings are the same.*

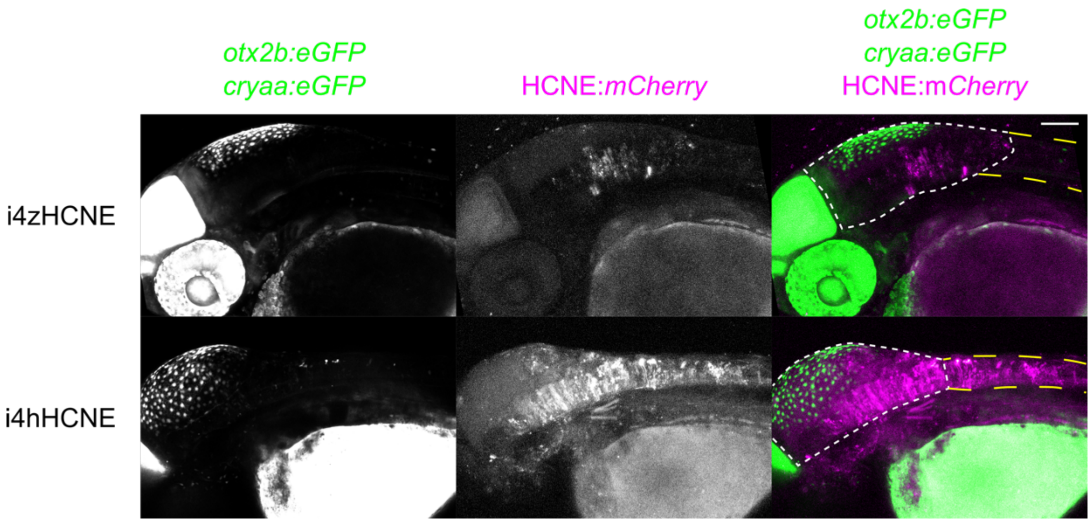

Supplemental Figure S29. Hnf1ba HCNEs from human and zebrafish both drive expression in the hindbrain. *48 hpf Tg(otx2b:EGFP) embryos injected with reporters i4zHCNE (top) and i4hHCNE (bottom). Left is otx2b:EGFP and cryaa:EGFP, middle is reporter with basal promoter E1b driving mCherry expression, and right is overlay of eGFP (green) and mCherry (magenta). 27 confocal z-slices were merged by z-projection based on maximum intensity. Embryos are orientated laterally. Dorsal is up, and anterior is on the left. Short white dashed line marks hindbrain, long yellow dashed line marks neural tube. Scale bar = 100mm. Embryos are representative of embryos that have the reporter construct incorporated (have cryaa:EGFP expression in lenses) and show expression of the HCNE in the hindbrain.*

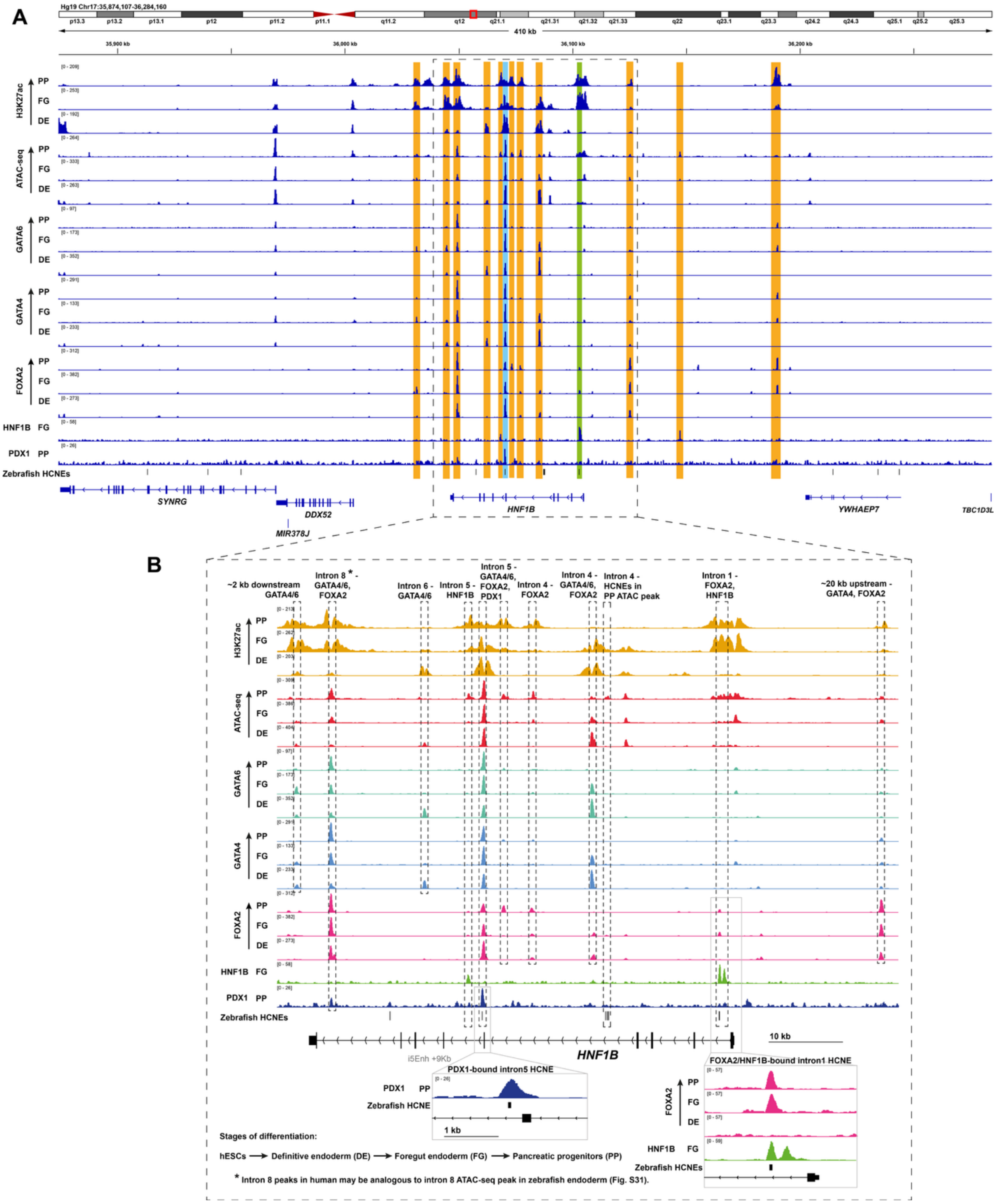

Supplemental Figure S30. Functional genomics analysis of the broader HNF1B genomic neighbourhood indicates binding of key pancreatic transcription factors at non-HCNE CRMs. *(A) ChIP-seq for H3K27ac, FOXA2, GATA4, GATA6, HNF1B and PDX1, and ATAC-seq data are shown. Orange boxes indicate peaks of interest that lack HCNEs (≥70% homology across ≥30 alignment columns between human and zebrafish genomes). The green box indicates an intron 1 HCNE exhibiting HNF1B binding that was not examined since the orthologous zebrafish HCNE lacked a significant ATAC-seq peak. The cyan box indicates the intron 5 HCNE bound by PDX1. Note that FOXA2 and GATA4/6 peak summits are adjacent rather than within the HCNE. ChIP-seq and ATAC-seq data are from (Lee et al. 2019). Note that the "i5Enh +9kb" enhancer region encompasses all of zebrafish exon 4 and intron 5 (Figure 7). These regions in the human genome contain GATA4/5, FOXA2 and PDX1 binding. (B) Zoomed image of boxed region from panel A with transcription factors binding at specific regions including HCNEs indicated. Note that the intron 4 HCNEs coincide with an ATAC-seq peak in pancreatic progenitors. DE = Definitive Endoderm; FG = Foregut endoderm; PP = Pancreatic Progenitors.*

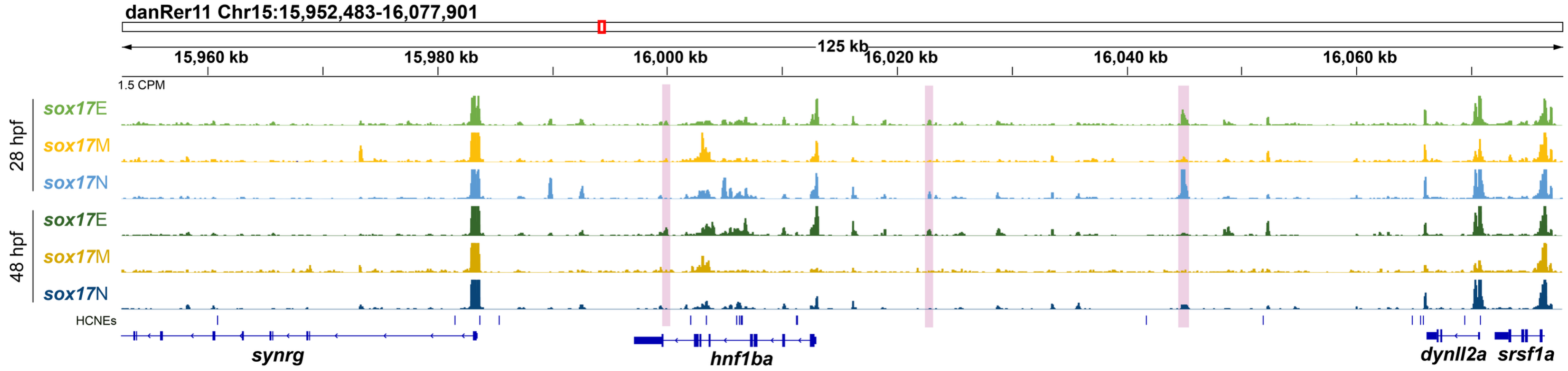

Supplemental Figure S31. Sox17E ATAC-seq analysis indicates potential CRMs upstream and in intron 8 of hnf1ba that could regulate endodermal expression. *ATAC-seq data as in Figure 7. Purple boxes indicate DARs showing significantly greater accessibility in sox17E over sox17M and/or sox17N populations that are not at the hnf1ba promoter, nor used in reporter assays (see Figure 7 and S28).*

Supplemental Table 16. Numbers of reads per sample passing filtering and used in analyses. *Note the high fractions of reads in peaks (FRiP), indicating high quality data.*

Library type	Stage	Sample	Replicate number	Number of mapped reads after filtering	Number of reads in peaks	Fraction of reads (FRiP) in peaks
**RNA-seq**	**28 hpf**	*sox17E*	1	30,045,054	NA	
			2	35,330,702		
		sox17N	1	32,917,422		
			2	40,449,434		
**ATAC-seq**	**28 hpf**	*sox17E*	1	14,120,110	5,988,722	0.42
			2	57,803,282	26,155,724	0.45
		sox17M	1	11,761,930	6,038,578	0.51
			2	45,117,334	19,166,820	0.42
		sox17N	1	115,198,342	61,324,177	0.53
			2	44,640,558	20,340,438	0.46
	**48 hpf**	*sox17E*	1	228,552,821	85,778,582	0.38
			2	75,691,821	39,963,901	0.53
		sox17M	1	70,325,093	18,754,448	0.27
			2	55,612,274	24,716,469	0.44
		sox17N	1	151,978,520	75,554,778	0.50
			2	86,888,006	55,262,315	0.64

Supplemental Table 17. Primers used in this study

Primers to amplify putative hnf1ba enhancers from genomic DNA

**Primer name**	**Sequence (5' to 3')**	
	**Forward**	**Reverse**
I5Enh +9Kb	ggggacaactttgtatagaaaagttg ACCAGCCAGTCAGTGTTACA	ggggactgctttttgtacaaacttg ACGACGTCAAGAGGAGAATGT
I5zHCNE	ggggacaactttgtatagaaaagttg TAGCAGCAATGCCATGTCAAC	ggggactgctttttgtacaaacttg AGCTACATGAAGATCTCGCC
I5hHCNE	ggggacaactttgtatagaaaagttg CAGCCAGTCGGTTTTACAGC	ggggactgctttttgtacaaacttg GAAGAGGGGGCTTGTGTCAA
I4 Enh +8Kb	ggggacaactttgtatagaaaagttg TCGCGTTCTCAGAGGTTGTA	ggggactgctttttgtacaaacttg GCTGAGTTTTGTTGTGTAGCG
I4 Enh +6Kb	ggggacaactttgtatagaaaagttg AGCAAAGAGGAGAGCAGTGA	ggggactgctttttgtacaaacttg GCAAACAGACAAGCCACTCA
I4 Enh +6-8Kb	ggggacaactttgtatagaaaagttg AGCAAAGAGGAGAGCAGTGA	ggggactgctttttgtacaaacttg GCTGAGTTTTGTTGTGTAGCG
I4zHCNE	ggggacaactttgtatagaaaagttg GCACTGCAATGAGTGGCTTG	ggggactgctttttgtacaaacttg GACCTTCTAATGGCGGCGAA
I4hHCNE	ggggacaactttgtatagaaaagttg CCAAACATCAGCACCTGAGC	ggggactgctttttgtacaaacttg CTGCCGACTAGAGCAAAGGG
Enh -3Kb	ggggacaactttgtatagaaaagttg GTGCACGCAACTCTAAGGTT	ggggactgctttttgtacaaacttg TGGGGTGTACTTAATTATGCTGA

Primers to switch eGFP for mCherry in reporter constructs

**Primer name**	**Sequence (5' to 3')**		**Use**
	**Forward**	**Reverse**	
eGFP	gacattaccgATGGT GAGCAAGGGCGA G	ggccgctttaCTTGTA CAGCTCGTCCAT GC	Amplify *eGFP* region from pENTR bas:EGFP

mCherry	GTGAGCAAGGGCGAGGATGACAACATGG	tgagtccggaTTACTTGTACAGCTCGTCC	Amplify *mCherry* region from pDEST tol2 pA *cryaa: mCherry*
pENTR baseGFP	gtacaagtaaTCCGGACTCAGATCTCGAG	tcatcctcgcCCTTGCTCACCATGGTGG	Amplify pENTR bas:EGFP backbone without *eGFP*
pDESTtol2pA cryaamCherry	gctgtacaagTAAAGCGGCCGCGACTCTAG	tgctcaccatCGGTAATGTCAGACCTGGTAAC	Amplify pDEST tol2 pA *cryaa:mCherry* backbone without *mCherry*

Primers to amplify E1b promoter for HiFi insertion into pENTR plasmid

Primer name	Sequence (5' to 3')	
	**Forward**	**Reverse**
E1b promoter HiFi	acaaaaaagcaggctcgcta CTCGACTCTAGAGGGTATATAATG	cttgctcaccatggtggcga TTTGCCAAAATGATGAGAC

Supplemental Methods

Zebrafish embryo dissociation for FACS

Embryos were dechorionated using 1 mg/ml pronase and deyolked in calcium free Ringer solution (0.5 M EDTA, 116 mM NaCl, 2.9 mM KCl, 5 mM HEPES). Deyolked embryos were dissociated in 20 mg/ml Collagenase (Sigma #C8176-25MG), 0.05% Trypsin with EDTA (Gibco #15400-054) in 1x Hank's Balances Salt Solution (HBSS, Gibco #14185-045) and homogenised using a pipette tip. The reaction was stopped by addition of foetal bovine serum to 5%, followed by dilution in HBSS+/+ (1X HBSS, 0.25% (w/v) BSA, 1 mM HEPES). Cells were pelleted and washed in HBSS +/+ buffer followed by filtration through a 70 µm cell strainer (Millex-GP). Cells were centrifuged for 5 minutes at 750 × g and re-suspended in HBSS+/+ buffer and transferred to polypropylene tubes pre-coated with 5% FBS in 1x PBS.

ATAC-seq

50,000 sorted cells per sample were used for OMNI-ATAC-seq using adapted methods outlined by (Buenrostro et al. 2015) and (Corces et al. 2017). All steps were conducted on ice or in centrifuges at 4°C. Cells were washed in ice cold 1x PBS prior to centrifugation at 500 × g for 5 minutes. Cell pellets were resuspended with cell lysis buffer (10mM Tris-HCl pH 7.5, 10mM NaCl, 3mM $MgCl_2$, 0.1% (v/v) NP-40, 0.1% (v/v) Tween-20, 0.01% (v/v) Digitonin) and were spun down at 4°C 500 × g for 10 minutes. Pelleted nuclei were resuspended in

transposition reaction mix (1X Tagment D buffer (Illumina #15027866), 5% (v/v) Tagment DNA Enzyme 1 (Nextera), PBS, 0.1% (v/v) Tween-20, 0.01% (v/v) Digitonin). Transposition reaction was incubated shaking at 1,000 rpm at 37°C for 30 minutes. DNA was isolated using Qiagen MinElute PCR Purification Kit and eluted using 22μl elution buffer. The remaining ATAC-seq library preparation was performed as described previously (Buenrostro et al. 2015). Bioanalyzer High Sensitivity DNA Analysis kit (Agilent) was used to test the quality of the purified libraries and ensure fragment size periodicity with intervals of around 200 bp. Bioanalyzer results were used to estimate concentrations and inform dilutions to be conducted before sending samples for sequencing. Libraries were sequenced by the Genomics Facility at the University of Warwick with Illumina NextSeq 500 using the High Output Kit v2.5 (FC-404-2002), and by Novogene (UK) Company Limited using NovaSeq 6000-S4-type flow cell. Each sample used different barcoded reverse primers, allowing for the samples to be multiplexed for sequencing.

Embryo imaging and image processing

Embryos were imaged using a Nikon ECLIPSE Ni, Zeiss Axio Zoom.V16 or Zeiss 980 confocal microscope. Images were analysed and processed using ImageJ/Fiji (Schindelin et al. 2012) version 2.9.0/1.52t. Processing included: adjusting the brightness and contrast setting, adding scale bars, creating and saving composite images, changing LUTs, and applying math log function to help deal with saturation. Bio-Formats Plugins for ImageJ (Linkert et al. 2010) was used to import files. Temporal-Colour Code was used to generate a 2D image from a stack of widefield fluorescent images, with each focal slice colour coded based on the Ice LUT. 3Dscript (Schmid et al. 2019), an ImageJ/Fiji plugin, was used to render 3D confocal images. Independent transparency was used as the rendering algorithm.

References

Buenrostro JD, Wu B, Chang HY, Greenleaf WJ. 2015. ATAC-seq: A Method for Assaying Chromatin Accessibility Genome-Wide. *Curr Protoc Mol Biol* **109**: 21 29 21-21 29 29.

Corces MR, Trevino AE, Hamilton EG, Greenside PG, Sinnott-Armstrong NA, Vesuna S, Satpathy AT, Rubin AJ, Montine KS, Wu B. 2017. An improved ATAC-seq protocol reduces background and enables interrogation of frozen tissues. *Nature methods* **14**: 959-962.

Farrell JA, Wang Y, Riesenfeld SJ, Shekhar K, Regev A, Schier AF. 2018. Single-cell reconstruction of developmental trajectories during zebrafish embryogenesis. *Science* **360**.

Gupta S, Stamatoyannopoulos JA, Bailey TL, Noble WS. 2007. Quantifying similarity between motifs. *Genome Biol* **8**: R24.

Lee K, Cho H, Rickert RW, Li QV, Pulecio J, Leslie CS, Huangfu D. 2019. FOXA2 Is Required for Enhancer Priming during Pancreatic Differentiation. *Cell Rep* **28**: 382-393 e387.

Linkert M, Rueden CT, Allan C, Burel JM, Moore W, Patterson A, Loranger B, Moore J, Neves C, Macdonald D et al. 2010. Metadata matters: access to image data in the real world. *J Cell Biol* **189**: 777-782.

Miguel-Escalada I, Maestro MA, Balboa D, Elek A, Bernal A, Bernardo E, Grau V, Garcia-Hurtado J, Sebe-Pedros A, Ferrer J. 2022. Pancreas agenesis mutations disrupt a lead enhancer controlling a developmental enhancer cluster. *Dev Cell* **57**: 1922-1936 e1929.

Ou J, Wolfe SA, Brodsky MH, Zhu LJ. 2018. motifStack for the analysis of transcription factor binding site evolution. *Nat Methods* **15**: 8-9.

Schindelin J, Arganda-Carreras I, Frise E, Kaynig V, Longair M, Pietzsch T, Preibisch S, Rueden C, Saalfeld S, Schmid B et al. 2012. Fiji: an open-source platform for biological-image analysis. *Nat Methods* **9**: 676-682.

Schmid B, Tripal P, Fraass T, Kersten C, Ruder B, Gruneboom A, Huisken J, Palmisano R. 2019. 3Dscript: animating 3D/4D microscopy data using a natural-language-based syntax. *Nat Methods* **16**: 278-280.

Sur A, Wang Y, Capar P, Margolin G, Prochaska MK, Farrell JA. 2023. Single-cell analysis of shared signatures and transcriptional diversity during zebrafish development. *Dev Cell* **58**: 3028-3047 e3012.
